# Roles of Msx2 in exogen control: modulating the stem cell niche during the transition from hair shedding to regeneration

**DOI:** 10.1016/j.jare.2025.09.040

**Published:** 2025-09-23

**Authors:** Qi Chen, Wen-Chien Jea, Ting-Xin Jiang, Ping Wu, Chi Zhang, Ji Li, Mingxing Lei, Cheng-Ming Chuong, Ya-Chen Liang

**Affiliations:** a Department of Dermatology, The First Affiliated Hospital of USTC, Division of Life Sciences and Medicine, University of Science and Technology of China, Hefei, Anhui 230001, China; b Department of Pathology, Keck School of Medicine, University of Southern California, Los Angeles, CA 90033, USA; c Department of Dermatology, Xiangya Hospital, Central South University, Changsha, Hunan 410008, China; d Key Laboratory of Biorheological Science and Technology of Ministry of Education & 111 Project Laboratory of Biomechanics and Tissue Repair, College of Bioengineering, Chongqing University, Chongqing 400044, China

**Keywords:** Exogen, Hair follicle stem cell, Niche, TGF-beta signaling, Hair regeneration

## Abstract

**Introduction:**

The exogen phase of the hair follicle cycle, during which club hairs are shed and new hairs emerge, represents a critical transitional step involving distinct structural remodeling and adhesive changes. Despite its fundamental importance, the molecular mechanisms orchestrating exogen remain largely unknown.

**Objective:**

To investigate the role of Msx2 in regulating hair anchoring, hair follicle stem cell (HFSC) lineage commitment, and extracellular matrix (ECM) remodeling during exogen.

**Methods:**

We employed *Msx2*-knockout (KO) mice, bulk RNA sequencing, and late exogen transcriptome comparisons to identify differentially expressed genes (DEGs) and disrupted pathways. Additionally, protein interaction assays were conducted for functional studies.

**Results:**

*Msx2* deficiency mice still form new bulges containing HFSCs for subsequent hair cycles, but this resulted in the failed anchoring of older bulges and club hairs, leading to aberrant hair retention and accelerated hair shedding. Transcriptomic analysis revealed widespread transcriptional reprogramming, particularly in ECM-related genes, with significant overlap between *Msx2*-KO HFSCs and late exogen DEGs. *Msx2*-KO mice exhibited downregulated adhesion molecules, disrupted HFSC niche integrity, increased keratinization, and aberrant differentiation in HFSC and hair germ cells. Mechanistically, MSX2 directly interacts with SMAD proteins to regulate TGF-β signaling, thereby modulating ECM gene expression and suppressing HFSC trans-epidermal differentiation.

**Conclusions:**

Our findings establish *Msx2* as a master regulator of exogen control, orchestrating hair shedding, HFSC niche stability, and ECM remodeling. This study provides new insights into hair follicle cycling dynamics and identifies Msx2 as a potential therapeutic target for promoting hair regeneration and mitigating alopecia phenotypes.

## Highlights

• *Msx2* deficiency impairs club hair anchoring and disrupts the exogen shedding process.
• HFSCs in *Msx2*-KO mice exhibit niche instability and abnormal epidermal differentiation.
• Loss of adhesion-related ECM genes contributes to defective hair follicle anchoring.
• Msx2 modulates TGF-β/Smad signaling to maintain HFSC identity and ECM homeostasis.
• The Msx2–ECM axis represents a promising target for hair regeneration therapies.

## Graphical abstract

**Figure:**
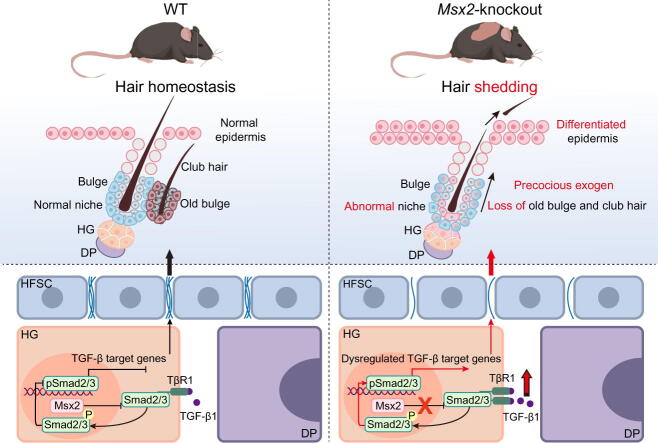

## Introduction

The hair follicle is a dynamic mini-organ that cycles through anagen (growth), catagen (regression), telogen (rest), and exogen (shedding) phases [1]. Exogen, marking the release of the club hair, is a distinct yet understudied transition critical for hair renewal [2], [3]. Although often viewed as an extension of telogen, it involves specific changes in adhesion and tissue remodeling [4], [5]. Dysregulation of exogen is implicated in hair loss disorders such as androgenetic alopecia (AGA) and telogen effluvium, but its molecular basis remains unclear [2], [6]. Several studies suggest that exogen is regulated by proteolytic activity and adhesion-related signaling, with roles proposed for matrix metalloproteinases, their inhibitors (e.g., TIMP3), and desmosome integrity [5], [7], [8]. Therefore, understanding the mechanisms of telogen club hair retention and the triggers of exogen may provide therapeutic insight into stabilizing hair loss, a process involving both club hair shedding and new hair emergence [7].

Several mouse models have been described in which the exogen phenomenon is altered, yet the underlying mechanism of club fiber shedding in *Msx2*-knockout (KO) mice remains unclear [8], [9]. *Msx2* is a pivotal transcription factor that modulates many biological processes by binding to specific DNA sequences [10]. It is prominently expressed in embryonic, bladder, and skin tissues, critically influencing the proliferation, migration, and differentiation of ectodermal neural crest cells during embryogenesis, as well as the functionality of adult stem cells [11]. Research has demonstrated that MSX2 interacts with the SWI/SNF complex, inhibiting the premature differentiation of progenitor cells and modulating the behavior of human trophoblast stem cells, which are essential for placental development and maturation [12]. Additionally, Msx2 is implicated in wound-induced hair neogenesis (WIHN), functioning through the induction of epithelial-mesenchymal transition (EMT) [13]. In *Msx2*-KO mice, evidence reveals disrupted hair cycling and progressive hair loss compared to wild-type (WT) counterparts. Furthermore, the mutant hairs exhibit increased susceptibility to detachment, suggesting potential impairments in cell adhesion [9]. Notably, downregulation of *Msx2* has been observed in patients with AGA, further emphasizing its importance in regulating the hair cycle [14]. Nonetheless, the precise molecular targets through which Msx2 exerts its regulatory effects on hair regeneration, particularly in telogen and exogen phases, remain to be definitively identified.

ChIP-qPCR analyses demonstrate a robust binding affinity of Smad2 to the promoter region of Msx2 [15]. This interaction suggests that Msx2 may facilitate the hematopoietic differentiation of human embryonic stem cells by modulating TGF-β signaling pathways. TGF-β signals originating from the dermal papilla (DP) initiate the phosphorylation of Smad in hair germ (HG) cells, which triggers the activation of dormant hair follicle stem cells (HFSCs) and fosters tissue regeneration [16]. Consequently, the potential involvement of Msx2 in regulating HFSCs through TGF-β signaling warrants further exploration.

In this study, our objective was to understand exogen control by leveraging the phenotype of *Msx2*-KO mice as a biological clue. While *Msx2*-KO mice were capable of forming new bulges containing HFSCs, club hairs from prior cycles remain unanchored, impairing proper shedding. Bulk RNA sequencing (RNA-seq) of *Msx2*-KO HFSCs revealed transcriptional alterations enriched in extracellular matrix (ECM) organization and cell adhesion genes. Comparative analysis with late-exogen datasets identified overlapping differentially expressed genes (DEGs), emphasizing the role of Msx2 in preserving HFSC niche structure under normal physiological conditions. Immunostaining and transcript profiling further confirmed reduced adhesion molecule expression and aberrant epidermal differentiation in HFSCs and HG cells. Mechanistically, MSX2 interacts with SMAD proteins to modulate TGF-β signaling, thereby regulating ECM-associated gene expression and suppressing HFSC trans-epidermal differentiation. This mechanism supports stem cell anchorage and ensures orderly follicular turnover during exogen transition. Collectively, our findings establish Msx2 as a pivotal regulator of exogen, essential for HFSC lineage fidelity and club hair anchoring. These insights uncover a novel mechanism of hair shedding control and identify Msx2 as a promising therapeutic target in hair regeneration and the control of alopecia.

## Materials and methods

### Ethics statement

All animal procedures were conducted in accordance with protocol #9473 approved by the Institutional Animal Care and Utilization Committee (IACUC) of the University of Southern California (USC, Los Angeles, CA, USA).

### Animal models

*Msx2*-KO mice were generated as previously described [9], [13], [17]. Briefly, a 9-kb thymidine kinase (TK) cassette was removed from the BamHI–SalI *Msx2* genomic fragment, and a pMC1-neo selection cassette was inserted at the NdeI site upstream of exon 2. The targeting construct was electroporated into embryonic stem cells, and correctly targeted clones were identified by genotyping. Chimeric mice were bred to obtain germline transmission. *Msx2* homozygous knockout mice were generated by intercrossing heterozygous animals. Hair cycle-matched C57BL/6J mice (Jackson Laboratory, Bar Harbor, ME) served as WT controls. Mice were maintained under a 12 h light/dark cycle at 21–26 ℃ and 30–70 % relative humidity in the USC animal facility.

### 5-bromo-2′-deoxyuridine (BrdU) administration

To visualize the transiently amplifying cells, mice were administered BrdU (B9285, Sigma) at 50 mg/kg via intraperitoneal injection. The samples were harvested seven days subsequent to the injection.

### Hematoxylin and Eosin (H&E) staining

Dorsal skins were collected, fixed in 4 % paraformaldehyde (PFA; HT501128, Sigma), paraffin-embedded, and sectioned at 8–10 μm. Sections were stained with hematoxylin (GHS 1–16, Sigma) and Eosin Y (HT1101-16, Sigma) for 1 min at room temperature (RT), then dehydrated with 95 % and 100 % ethanol, and fixed with xylene (6601, Thermo Scientific). Images were acquired using a Keyence microscope with a 20× objective lens and analyzed in ImageJ and Adobe Photoshop.

### Immunofluorescence and TUNEL staining

Paraffin sections were deparaffinized and rehydrated. Blocking solution including 2.5 % normal goat serum (G9023, Sigma), 1 % Bovine Serum Albumin (BSA; A3059, Sigma), and 0.1 % Tween-20 (P9416, Sigma) in PBS (MB1011, BioPioneer) was used to block sections for 1 h at RT. Primary antibodies diluted in 1 % PBT were applied overnight at 4 ℃. Sections were washed and incubated with Alexa Fluor 488 and 594-conjugated secondary antibodies (1:200, A11029 and A11072, Invitrogen) for 2 h. Nuclei were stained using 4′,6-diamidino-2-phenylindole (DAPI; 62248, Thermo Scientific). TUNEL staining was performed using the TUNEL assay kit (11684817910, Roche). As a positive control for TUNEL staining, adjacent skin sections were pretreated with 3 U/mL DNase I (10104159001, Roche) at RT for 10 min to induce DNA strand breaks.

The antibodies and dilutions were used as follows: FOXC1 (1:200, 8758, Cell Signaling), pSMAD2 (1:100, 3101, Cell Signaling), Cytokeratin 15 (1:200, MA1-90929, Thermo Scientific), Cytokeratin 1 (1:100, PA5-114755, Thermo Scientific), Cytokeratin 10 (1:100, 39–5300, Thermo Scientific), Cytokeratin 6 (1:500, PRB-169P, BioLegend), LEF1 (1:100, 2230S, Cell Signaling), Collagen VI (1:100, ab6588, Abcam), POSTN (1:100, ab14041, Abcam), FBLN1 (1:100, ab211536, Abcam), ANGPTL2 (1:100, 12316–1-AP, Proteintech), Collagen IV (1:200, ab6586, Abcam), PCNA (1:100, CBL407, Chemicon), F-ACTIN (1:100, M1210-2, Huabio), TGF-β1 (1:100, 69012-1-1g, Proteintech), and Caspase-3 (1:200, ab184787, Abcam).

### Multicolor RNA-In Situ Hybridization (RNAscope)

The 4 % PFA-Fixed Paraffin-Embedded sections were deparaffinized using xylene and 100 % Ethanol (470301-072, Ward’s science) for 10 min, twice. The reagents and probes used in this procedure were purchased from Advanced Cell Diagnostics (ACD, CA, USA). The slides were air-dried for 5 min at 60 ℃. Next, the entire section was covered with 3 % hydrogen peroxide (H_2_O_2_) for 10 min at RT and then placed in the boiling DEPC H_2_O for 15 s. Subsequently, submerge the sections into boiling retrieval reagents for 15 min. Immediately transfer the hot slide to DEPC H_2_O for 15 s, twice at RT. Wash slides in 100 % Ethanol for 3 min and air dry for 5 min at 60 ℃. Place the dried slides on the HybEZ™ Slide Rack and add 1–2 drops of Protease Plus. Incubate in the oven at 40 ℃ for 30 min. After washing the slides with DEPC H_2_O, mixed probes were added, and the slides were incubated in the oven for 2 h at 40 ℃. Wash slides with Wash Buffer at RT for 5 min. Add 1–2 drops of AMP 1-FL in the oven for 30 min at 40 ℃. Then wash and add 1–2 drops of AMP 2-FL in the oven for 30 min at 40 ℃. Then wash and add 1–2 drops of AMP 3-FL in the oven for 15 min at 40 ℃. Add 1–2 drops of HRP-C1/C3, Opal 520/570 (1:600 dilution in Multiplex TSA buffer), and HRP Blocker sequentially, and incubate for 15 min, 30 min, and 15 min at 40 ℃. Nuclei were stained using DAPI. Images were acquired by a Keyence microscope with LAS software. Figures were prepared using Adobe Photoshop.

The following probes and dilutions were used: Msx2 (1:150, 421851-C3) and Lhx2 (1:1, 485791).

### Fluorescence-Activated Cell Sorting (FACS)

HFSCs and epidermal stem cells (EpdSCs) were isolated from the dorsal skin of 6-month-old *Msx2*-KO mice, following previously described protocols [18]. Briefly, dorsal skin was excised and carefully trimmed to remove subcutaneous fat, muscle, and connective tissue. The tissue was placed dermis-side down in 0.25 % trypsin-EDTA solution (T4049, Sigma) and incubated overnight at 4 ℃ to facilitate epidermal separation. The following day, the epidermis was gently peeled away using forceps, and hair follicles were mechanically dissociated in cold PBS. The resulting cell suspension was neutralized by adding 0.05 mM Ca^2+^ medium, filtered sequentially through 70 µm and 40 µm cell strainers (352350 and 352340, Falcon), and centrifuged at 300×*g* for 5 min at 4 ℃. Cell pellets were resuspended in staining buffer (2 % Chelex-treated serum without calcium in 1X PBS) and incubated with fluorophore-conjugated antibodies: CD34-BV421 (562608, BD Horizon), Sca-1-FITC (11-5981-82, eBioscience), CD49f (integrin α6)-PE (12-0495-83, eBioscience). Drag5 (65-0880-92, eBioscience) was used to exclude dead cells. Sorting was performed on a BD FACSAria II (v8.0.2) equipped with Diva software at USC Flow Cytometry Core. Flow cytometry data were analyzed using Attune NxT and FlowJo software. CD34^+^CD49f^+^Sca-1^−^ cells were identified as HFSCs, and CD34^−^CD49f^+^Sca-1^+^ cells were defined as EpdSCs (Fig. S2A).

### Bulk RNA-seq analysis

For each condition, RNA was extracted from FACS-isolated hair HFSCs, EpdSCs, or HaCaT cells (with two or three replicates per group) using the AllPrep DNA/RNA Micro Kit (80284, Qiagen). Library preparation with the SMARTer Stranded Total RNA-Seq Kit v3 was performed at Novogene, Inc. (CA, USA), and RNAs were sequenced on Illumina NovaSeq 6000. RNA-seq data for hair follicles of young and aged EpdSCs (GSE185087) were downloaded from the Gene Expression Omnibus database. Analysis of RNA-seq data was performed by Partek Flow Bioinformatics Software (licensed by USC). Reads were aligned to the mouse reference genome (GRCm38/mm10) using the STAR aligner with default parameters. Gene annotation was based on GENCODE release M25. Differential gene expression analysis was conducted using Gene-specific Analysis (GSA). Genes with an absolute fold change (FC) > 2 and false-discovery rate (FDR) < 0.05 were considered significantly different between the two groups under comparison. Data visualization and enrichment analyses were performed using the online platform Bioinformatics.com.cn.

### scRNA-seq analysis

The scRNA-seq data for telogen and anagen hair follicles were downloaded from accession code GSE90848. Cell clusters “Telo-HG”, “Ana-HG”, and “Telo-HFSCs” were reanalyzed and visualized using a 2-dimensional Uniform Manifold Approximation and Projection (UMAP) algorithm (Fig. S1A).

### Omni-ATAC-seq library preparation and analysis

Omni-ATAC-seq libraries were prepared following the published protocol [19]. Briefly, cells were resuspended in lysis buffer (10  mM Tris-HCl, pH = 7.4, 10  mM NaCl, 3  mM MgCl_2_, 0.1 % NP40, 0.1 % Tween-20, and 0.01 % Digitonin) for 5 min at 4 ℃. After lysis, ATAC-resuspension buffer (same composition without detergents, plus 0.1 % Tween-20) was added, and nuclei were pelleted by centrifugation at 2500 r.p.m. at 4 ℃ for 5 min. Nuclei were then resuspended in the transposition reaction mix containing Tn5 transposase (15027866, Illumina) and incubated at 37 ℃ for 30 min. DNA was purified using the Zymo DNA Clean & Concentrator kit (D4014). Samples were PCR-amplified using NEBNext High-Fidelity PCR Master Mix (M0541, New England Biolabs, MA), and 1.25  μM of custom Nextera primers, using the following program: 72 ℃ for 5 min, 98 ℃ for 30 s, followed by 5 cycles (98 ℃ for 10 s, 63 ℃ for 30 s, 72 ℃ for 1 min). After the initial 5 cycles, a 5 μl aliquot was removed, combined with SYBR Green I (S7563, Invitrogen), and subjected to quantitative PCR (qPCR) to determine the optimal number of additional cycles. The final number of cycles was defined based on reaching one-fourth of the maximum fluorescence intensity. Libraries were then amplified accordingly and purified. Final DNA was eluted in 15 μl of 10 mM Tris-HCl (pH = 8.0).

For sequencing, raw reads were trimmed using Trimmomatic v0.30 to remove adapters, low-quality reads, and short fragments (<50 bp). Clean reads were aligned to the mouse genome (GRCm38/mm10) using BWA-MEM. Mitochondrial reads were excluded. Peaks were called using MACS2 with the following parameters: q-value cutoff <0.01, no shifting model, shift size = 100, extension size = 200, and duplicate reads retained (keep dup all). Output files included both narrowPeak and bedGraph formats. IGV (v2.17.0) was used to visualize the peaks. Within Partek Flow, significant ATAC-seq peaks were annotated and categorized as promoter-proximal (±1500 bp from the transcription start site [TSS]) or distal enhancer regions. By integrating RNA-seq expression profiles, we further classified these peaks into active or repressive promoters and enhancers based on the transcriptional activity of associated genes.

### Lentivirus induction of Msx2 overexpression in keratinocytes

Human keratinocytes (HaCaT cells; CL-0090, Procell) were cultured in Minimum Essential Medium (MEM; PM150411, Procell) supplemented with 15 % fetal bovine serum (FBS; PM00011, Proteintech) and 1 % penicillin–streptomycin (PB180120, Procell) at 37 ℃ in a humidified 5 % CO_2_ incubator. Full-length mouse *Msx2* with a C-terminal V5-His tag was cloned into the CSII-CMV-MCS-IRES2-Bsd lentiviral vector (Juyan Biotechnology Co., Ltd., Hefei, China). Lentiviruses were produced by co-transfecting this construct with packaging plasmids (pRSV-Rev, pMD2. G, and pMDLg/pRRE; Addgene) into 293 T cells (CRL-3216, ATCC) using polyethylenimine (PEI; Polysciences Inc.). Viral supernatants were collected and concentrated with Lenti-X Concentrator (Takara Bio). Primary human keratinocytes were seeded in six-well plates and transduced with 150 μL of concentrated lentivirus in the presence of 4 μg/mL polybrene (TR1003, Sigma). After 24 h, the medium was replaced, and cells were cultured for another 48–72 h before selection with 2 μg/mL puromycin (ST551, Beyotime). Transduction efficiency was assessed by Western blot for V5-tagged *Msx2*, with over 80 % of cells showing positive expression.

### Co-immunoprecipitation (Co-IP)

To assess the interaction between MSX2 and SMAD2, co-IP assays were performed using HaCaT cells (CL-0090, Procell) and *Msx2*-overexpressing (OE) cells. After removing the culture medium, cells were washed twice with cold PBS and lysed in IP lysis buffer (87787, Thermo Scientific) at 4 °C for 3 h. Lysates were centrifuged at 12,000×*g* for 10 min at 4 °C. The supernatant was collected and incubated overnight at 4 ℃ with either anti-MSX2 (sc-365232, Santa Cruz), anti-SMAD2 (sc-393312, Santa Cruz), or isotype-matched control IgG (sc-515946, Santa Cruz). Immune complexes were captured using 30 μL of Protein A/G PLUS-Agarose beads (sc-2003, Santa Cruz) for 8 h at 4 ℃ with gentle rotation. Beads were then extensively washed with lysis buffer, and the bound proteins were eluted by boiling in SDS sample buffer for 10 min. Eluates were analyzed by SDS-PAGE followed by immunoblotting (IB) with anti-MSX2 and anti-SMAD2 antibodies.

### Enzyme-linked immunosorbent assay (ELISA)

Culture medium from vector control (VE) or OE cells was collected and centrifuged. The resulting supernatants were subjected to ELISA using a human TGF-β1 kit (abs552208, Absin) according to the manufacturer’s instructions.

### Western blot

Total protein lysates from VE or OE cells, treated with or without TGF-β1 (69012-1-1g, Proteintech), were separated by SDS-PAGE. Electrophoresis was performed at 90 V through the stacking gel and at 150 V through the separating gel until the bromophenol blue dye front reached approximately 1 cm from the bottom. Proteins were then transferred onto a PVDF membrane (IPVH00010, Merck) at a constant current of 300 mA for 30 min. Membranes were blocked with 5 % non-fat milk in TBST for 30 min at RT. After blocking, membranes were incubated overnight at 4 ℃ with primary antibodies in blocking buffer under gentle agitation. Following primary incubation, membranes were washed and incubated with appropriate HRP-conjugated secondary antibodies for 30 min at RT. Protein bands were visualized using enhanced chemiluminescence (ECL, Bio-Rad), and band intensities were quantified using ImageJ. Target protein levels were normalized to GAPDH.

The antibodies and dilutions were used as follows: SMAD2/3 (1:2000, 8685, Cell Signaling), pSMAD2 (1:2000, 3108, Cell Signaling), Cytokeratin 10 (1:1000, sc-53252, Santa Crutz), GAPDH (1:1000, 60004-1-Ig, Proteintech).

### Cell Counting Kit-8 (CCK-8) test

Cell viability was assessed by CCK-8 assay (abs50003, Absin). After 24 h of cultivation, the medium was replaced with 10 % CCK-8 reagent in each well. Following incubation, absorbance of each well at 450 nm was detected with a microplate reader (BioTek, USA).

### Transwell assay

A Transwell chamber pre-coated with Matrigel (Corning) was used to assess cell invasion in vitro. In brief, 200 μL of cell suspension was added to the upper chamber, while the lower chamber was filled with medium containing 5 % FBS as a chemoattractant. After 24 h of incubation, the cells that had invaded through the membrane were fixed and stained with 0.1 % crystal violet (G1064, Solarbio). Stained cells were imaged under an inverted optical microscope. Five random fields were selected for counting, and the average number of invaded cells was calculated.

### Statistical analysis

Statistical analyses were performed with GraphPad Prism 9.5.1. For all in vivo experiments, a minimum of three biological replicates (individual mice) were used per group, unless otherwise indicated. For cell-based assays and molecular analyses, experiments were repeated independently at least three times, with results reported as mean ± standard deviation (SD). Two-group comparisons were analyzed using two-tailed unpaired Student’s t-tests. Multiple comparisons were assessed using one-way ANOVA with Dunnett’s post hoc tests. *A p-*value < 0.05 was considered statistically significant.

### Data availability

The next-generation sequencing datasets generated during this study are available in the NCBI Gene Expression Omnibus (GEO). Bulk RNA-seq data have been deposited under accession number GSE303779, and ATAC-seq data under accession number GSE303778. Both datasets are publicly accessible. All other data supporting the findings of this study are available from the corresponding author upon reasonable request.

## Results

### Msx2 is dynamically expressed in HFSC and its niche during different phases of hair cycling

**Fig. 1:**
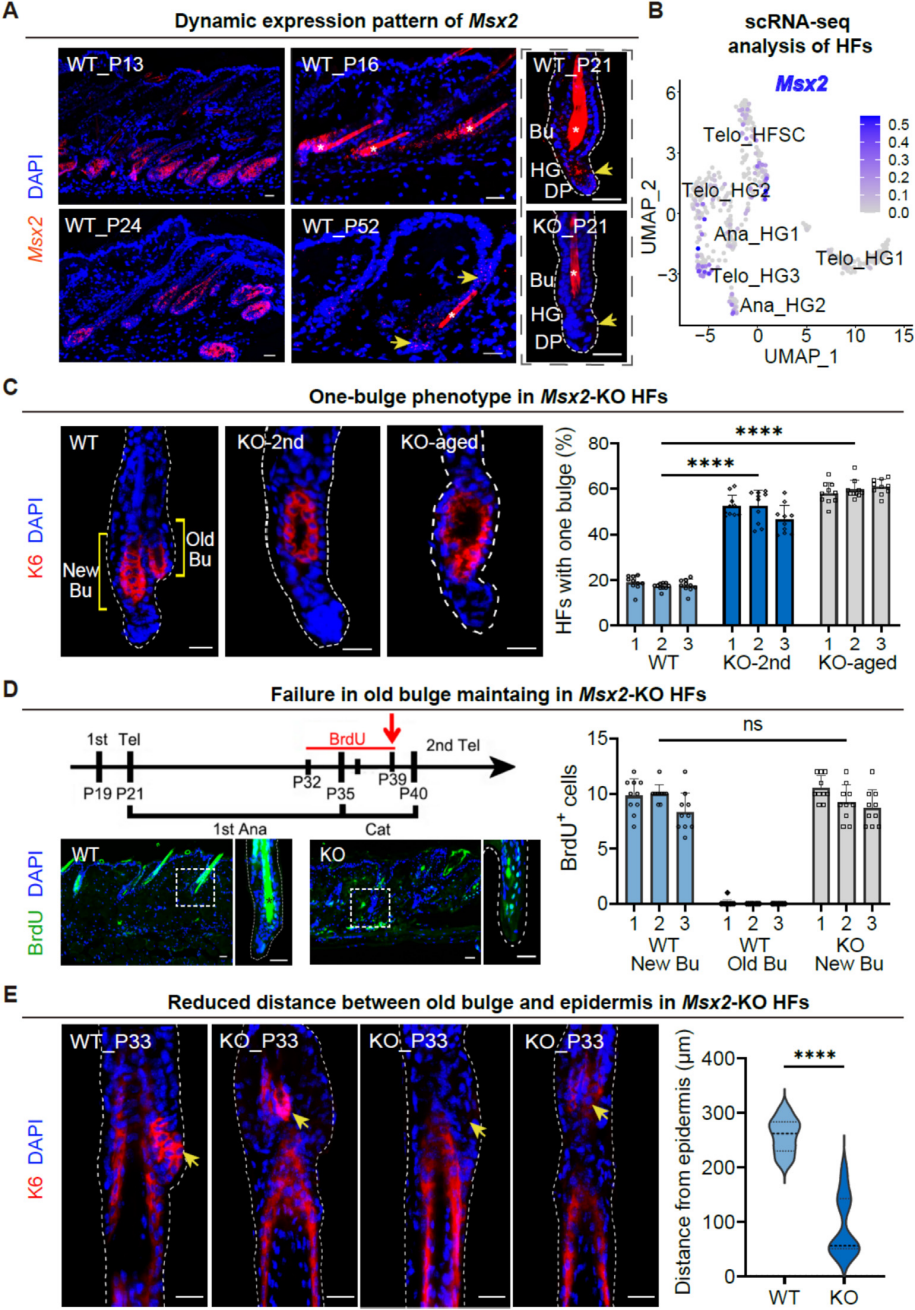
***Msx2*****deficiency disrupts maintenance of the old bulge during exogen.** (A) RNAscope reveals dynamic *Msx2* expression in hair follicles (HFs) across the hair cycle: anagen (P13, upper left; P24, lower left), catagen (P16, upper middle), and telogen (P21, right; P52, lower middle). Scale bars, 30 μm. Asterisks denote a nonspecific signal in the hair shaft. (B) UMAP projection of scRNA-seq data demonstrates *Msx2* expression within specific HF cell clusters, with most enrichment in telogen hair germ (Telo_HG) populations. (C) Immunofluorescence staining of K6 in the inner bulge (left) and quantification analysis of K6^+^ cells per HF (right). Scale bars, 30 μm. *N* = 3, ****p < 0.0001. (D) Schematic of BrdU pulse-chase experiment to track label-retaining cells (LRCs) from the anagen outer root sheath (ORS) to the telogen bulge (Bu). Scale bars, 30 μm. *N* = 3; ns, not significant. Asterisks denote a nonspecific signal. (E) Bulge positioning during the first anagen. *Msx2*-KO HFs exhibit loss of K6 in the bulge region and proximal displacement toward the epidermis (arrows). Scale bars, 30 μm. *N* = 3, ****p < 0.0001.

In our effort to unravel the regulatory mechanism within the hair follicle, early findings suggested a critical role for Msx2 in modulating the transition from transit-amplifying (TA) cells to precortical cells [9]. This observation prompted a deeper investigation into the spatiotemporal expression and functional significance of Msx2 throughout the hair cycle. To achieve this, we systematically analyzed the dynamic expression patterns of *Msx2* across distinct phases of the hair cycle using samples isolated from WT mice. Utilizing the RNAscope technology, we observed a robust expression of *Msx2* mRNA across the epithelial compartments at postnatal day 13 (P13), including the progenitors of the lower outer root sheath (ORS), inner root sheath (IRS), and matrix cells (Fig. 1A, upper left), corroborating previous studies [9]. As the hair cycle progressed into catagen (P16), *Msx2* was notably present within the regressing epithelial strand (Fig. 1A, upper middle). During telogen (P21), *Msx2* expression diminished, yet remained detectable primarily in the HG, as well as the bulge, inner bulge, and DP (Fig. 1A, upper right). At the threshold of the subsequent anagen (P24), *Msx2* expression rebounded and extended into the hair matrix cells and IRS of the hair follicles (Fig. 1A, lower left). In the second telogen stage (P52), the expression pattern of *Msx2* within the hair follicles persisted and remained high in HG, inner bulge, and DP. Moreover, *Msx2* was traceable in sparse cells within the infundibulum and the inter-follicular epidermis (Fig. 1A, lower middle). By contrast, the expression of *Msx2* was absent in *Msx2*-KO mice (Fig. 1A, lower right). By analyzing published scRNA-seq data from mouse skin, we discerned that *Msx2* expression was evident in telogen (Telo)-HG, anagen (Ana)-HG, and Telo-HFSC populations (Fig. 1B, Fig. S1B) [20]. These findings suggest a specialized role for Msx2 in governing HG and bulge HFSC functions.

We next employed *Msx2*-KO mice for further investigations. Compared to their WT counterparts, KO mice exhibited alopecia in the head skin at P12, suggesting a shortened anagen phase. Conversely, the telogen phase was significantly prolonged in KO mice, spanning from P18 to P28 (Fig. S1C), underscoring the pivotal role of Msx2 in the regulation of hair cycling. Immunostaining of the dorsal skin during the second telogen phase revealed no significant alterations in the localization of Keratin 6 (K6) within the inner bulge layer (Fig. 1C, left). Interestingly, while WT hair follicles displayed a distinct two-bulge structure, *Msx2*-KO hair follicles predominantly exhibited a single-bulge phenotype, even in aged mice (Fig. 1C, middle and right).

To ascertain whether *Msx2*-KO hair follicles were incapable of forming a new bulge or prematurely lost their original bulge, we employed BrdU to label the lower ORS cells in late anagen, a period when HFSCs are predominantly quiescent [21]. Typically, the new bulge arises partly from these BrdU-labeled ORS cells that survive the ensuing catagen, while the old bulge remains unlabeled [22]. In KO hair follicles, the single bulge exhibited BrdU immunofluorescence staining similar to that of WT counterparts, indicating its recent formation (Fig. 1D). Based on the published literature, it was shown that the conditional knockout of *Forkhead box C1* (*Foxc1*) could also result in a one-bulge structure since the second telogen [21]. Immunofluorescence assessment revealed that KO hair follicles maintained normal FOXC1 expression, implying that the loss of the old bulge was not attributable to the absence of *Foxc1* under *Msx2*-KO conditions (Fig. S1D).

To further investigate whether the old bulge underwent aberrant changes prior to the second telogen, we tracked the status of the old bulge and club hair during the preceding anagen phase. In WT hair follicles, the old bulge was situated adjacent to the newly developing hair follicle. By contrast, KO hair follicles displayed residual old bulge structures abnormally positioned closer to the epidermis and separated from the new bulge and associated club hair (Fig. 1E). These findings collectively suggest that while *Msx2*-KO hair follicles can generate a new bulge, they fail to preserve the structural integrity of the old bulge and club hair throughout the hair cycle. The shedding of club hair is closely associated with the exogen phase of the hair cycle. Previous studies comparing DEGs between early and late exogen stages revealed that *Msx2* expression is downregulated during the late exogen stage [7], indicating its potential critical role in regulating hair shedding.

### Impaired ECM deposition around HFSC niche is observed in Msx2-KO telogen and exogen HFs

**Fig. 2:**
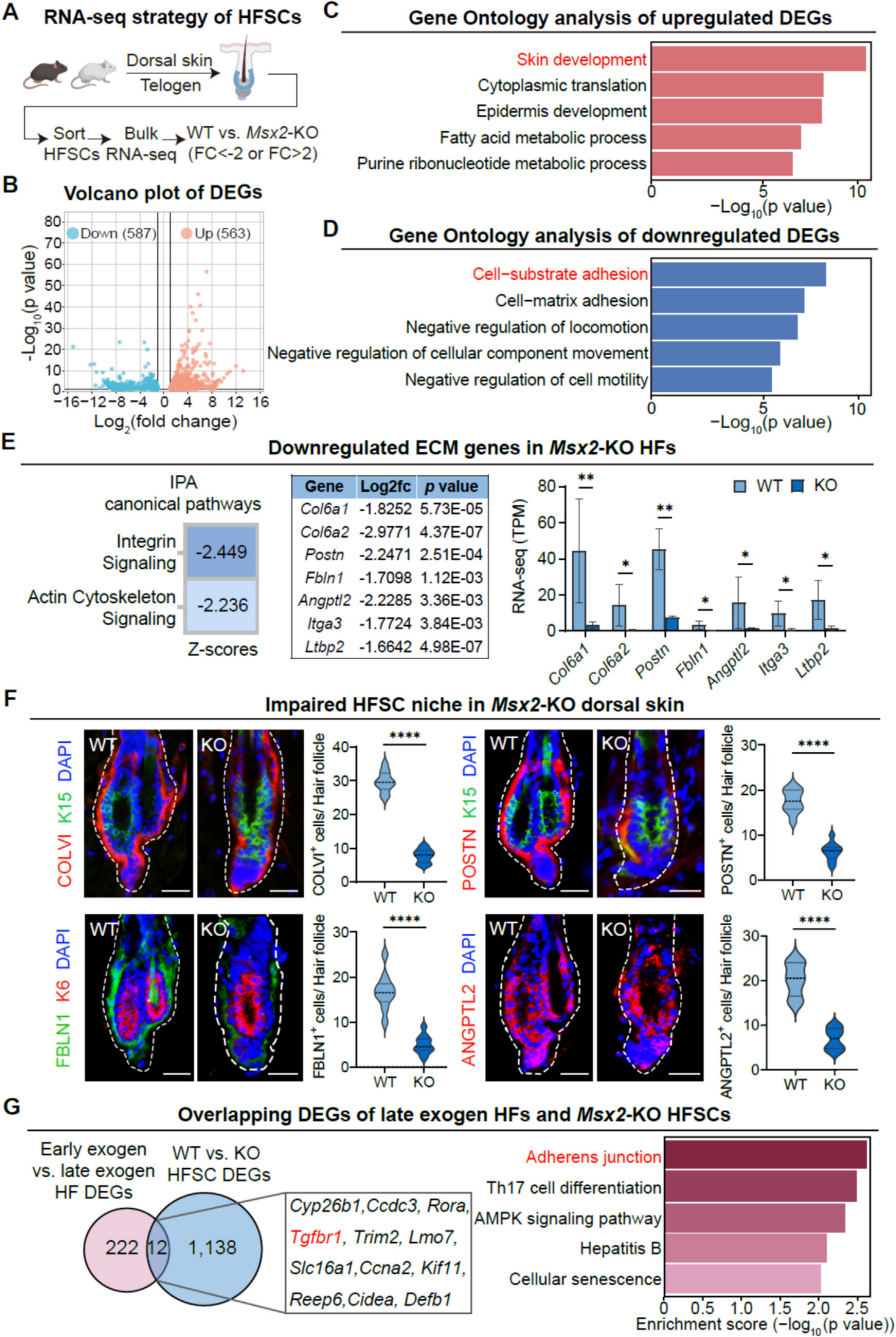
***Msx2*****deficiency impairs ECM deposition within the HFSC niche.** (A) Experimental workflow for identifying differentially expressed genes (DEGs) through bulk RNA-seq of sorted telogen HFSCs from WT and *Msx2*-KO mice. (B) Volcano plot displaying DEGs (adjusted *p* < 0.05). (C) Gene Ontology (GO) enrichment of upregulated DEGs identifies terms associated with skin development. (D) GO enrichment of downregulated DEGs highlights cell-substrate and matrix adhesion. (E) Ingenuity Pathway Analysis (IPA) of canonical pathways (left), key downregulated niche genes (middle), and their relative RNA expression levels identified through RNA-seq analysis (right). *N* = 2, **p < 0.01, *p < 0.05. (F) Immunofluorescence staining and quantification of ECM/adhesion molecules (COLVI, POSTN, FBLN1, and ANGPTL2) in the second telogen. Scale bars, 30 μm. *N* = 3, ****p < 0.0001. (G) Venn diagram of DEGs between early/late exogen HFs and WT/KO HFSCs (left); KEGG analysis of overlapping genes (right).

To uncover the molecular underpinnings of HFSC function during the critical transition from telogen to anagen, we employed FACS to meticulously isolate HFSCs, encompassing both bulge stem cells and HG cells, from WT and *Msx2*-KO mice (Fig. 2A, Fig. S2A). These cells were subsequently subjected to a comprehensive transcriptome analysis via RNA-seq. Our analysis revealed that, akin to their WT counterparts, HFSCs from KO mice did not exhibit significant deviations in the expression of established HFSC markers, such as Keratin 15 (K15) and LIM homeobox 2 (Lhx2) (Fig. S2B and Fig. S2C). By leveraging our RNA-Seq datasets, we curated a list of 18 HFSC signature genes and charted their expression patterns in both WT and KO HFSCs (Fig. S2C). Moreover, the quantitative assessment of HFSCs revealed no substantial disparity between the two groups (Fig. S2D) [23]. Collectively, these observations suggest that Msx2 is not required to establish the HFSC identity during its quiescent phase.

To delineate the primary transcriptional repercussions of *Msx2* ablation, we conducted a comparative analysis of the global gene expression profiles between HFSCs of WT and *Msx2*-KO mice. This analysis yielded 563 upregulated and 587 downregulated DEGs, with a statistical significance threshold of *P*adj (adjusted p-value) < 0.05 (Fig. 2B). Of note, the Gene Ontology (GO) term enrichment analysis of the RNA-seq data highlighted a significant enrichment of terms related to cell adhesion, cell motility, and ECM components within the downregulated genes (Fig. 2D). Among the downregulated genes, *Col6a1, Col6a2, Postn, Fbln1, Angptl2, Itga3, and Ltbp2* were identified as integral constituents of the HFSC niche, as previously reported (Fig. 2E) [24], [25].

To substantiate our RNA-seq findings, we proceeded to examine the expression of these pivotal cell adhesion molecules and ECM components. During the second telogen phase, we performed colocalization immunofluorescence using K15 or K6 antibodies alongside ECM markers including COLVI, POSTN, FBLN1, and ANGPTL2. These markers were detected near HFSCs, thereby confirming their role as ECM components of the HFSC niche. In stark contrast, KO HFs displayed a marked reduction in the levels of these adhesion molecules (Fig. 2F). Additionally, a comparison of early and late exogen stage DEGs with our identified genes revealed 17 overlapping genes [7], 12 of which showed consistent results. These genes were predominantly enriched in adherens junction, suggesting that they may play a crucial role in regulating exogen and telogen transitions (Fig. 2G). These findings provide strong evidence for aberrant ECM deposition in the HFSC niche of *Msx2*-KO hair follicles, highlighting the critical role of Msx2 in the regulation of telogen, which may contribute to the onset of hair loss.

### Msx2 deficiency leads to enhanced apoptosis and an aging-like phenotype in the skin

**Fig. 3:**
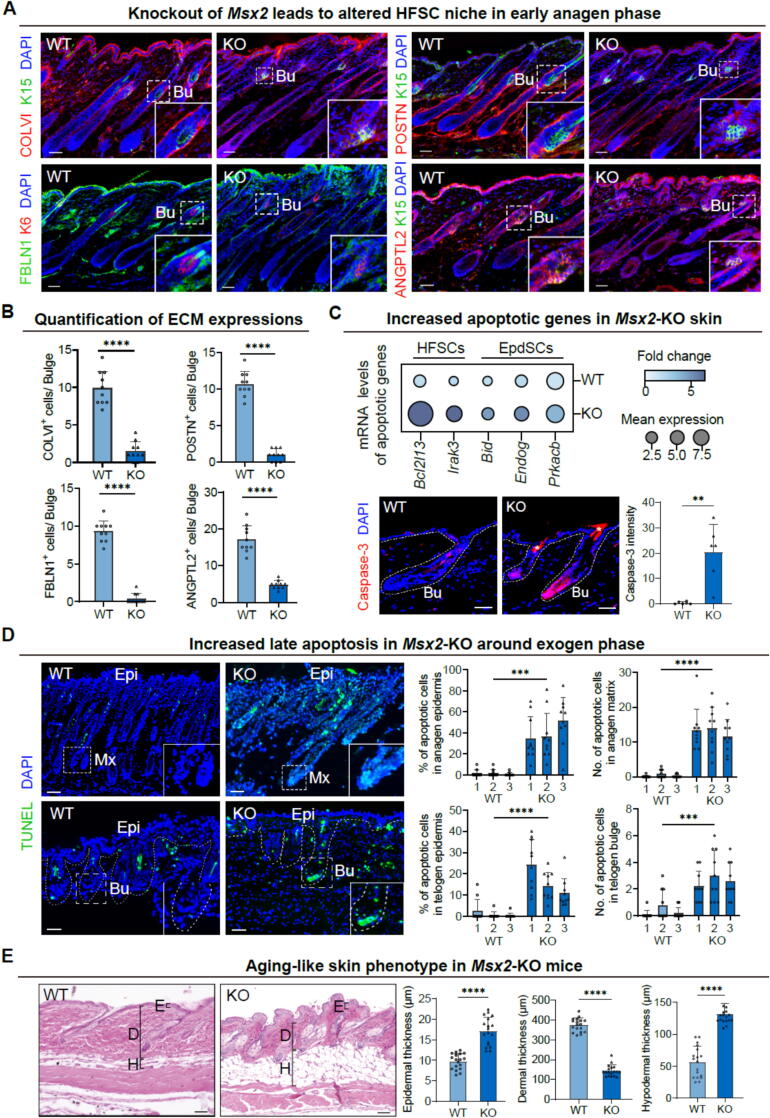
**Increased apoptosis and ECM defects in*****Msx2*****-KO mice during the anagen phase.** (A) Immunofluorescence staining shows decreased adhesion molecules in mutant bulge (Bu) regions during the early anagen. Scale bars, 30 μm. (B) Quantitative analysis of positive cells. *N* = 3, ****p < 0.0001. (C) Relative RNA expression levels of apoptotic markers identified through RNA-seq analysis (top); immunofluorescence staining of cleaved caspase-3 (lower left) with quantification of relative signal intensity (lower right). Scale bars, 30 μm. *N* = 3, **p < 0.01. Asterisks denote a nonspecific signal in the hair shaft. (D) TUNEL assay shows apoptosis in matrix (Mx), bulge (Bu), and epidermis (Epi) (left) with quantification (right). Scale bars, 30 μm. *N* = 3, ****p < 0.0001, ***p < 0.001. (E) Comparative histological analysis of telogen skin from age-, sex-, and strain-matched *Msx2*-KO and WT littermates. Scale bars, 30 μm. *N* = 3, ****p < 0.0001. E, epidermis; D, dermis; H, hypodermis.

Both exogen and telogen represent non-proliferative phases of the hair follicle cycle, with exogen beginning as telogen concludes [5]. Building upon our findings related to the abnormal HFSC niche in telogen, we extended our investigation into the early anagen phase, a critical period when exogen predominantly occurs in most hair follicles [8]. Our analysis revealed a distinct absence of ORS (K15 or K6) expression surrounding KO hair follicles, indicating their transition into the exogen phase. Furthermore, our analysis indicated a significant decline in the expression of essential adhesion proteins within the bulge compartment of mutant hair follicles (Fig. 3A and B). Transcriptomic analysis of HFSCs and EpdSCs uncovered a coordinated upregulation of apoptosis-associated genes (Fig. 3C). Immunofluorescence staining demonstrated increased cleaved caspase-3 expression in KO HFSCs (Fig. 3C). A subsequent examination of late apoptosis levels using the TUNEL assay revealed a pronounced increase in TUNEL-positive cells within the matrix, bulge region, and epidermis of *Msx2*-KO mice (Fig. 3D and Fig. S3A). Hence, loss of *Msx2* disrupts the skin ECM in the HFSC compartment, resulting in weakened cell–cell adhesion and greater vulnerability to apoptosis, which may ultimately lead to the absence of club hairs.

We then analyzed histological changes in age-, sex-, and strain-matched telogen skin. Notably, *Msx2*-KO hair follicles exhibited a disorganized arrangement, accompanied by enlarged sebaceous glands and increased epidermal thickness compared to young WT littermates. Additionally, the dermal layers of young mutant skin displayed aberrant characteristics, such as reduced dermis thickness and increased hypodermal thickness (Fig. 3E). In addition, collagen IV showed disorganized distribution and decreased expression at the interface between the HG and DP in *Msx2*-KO skin [26] (Fig. S3B). These observations bear resemblance to the histological features observed in aged WT mice [27]. Research has shown that aging is associated with ECM deterioration in both mice and humans, which may be a contributing factor to the impaired hair regenerative capacity observed in older mice [28], [29]. These findings suggest that loss of *Msx2* influences cell adhesion composition, which may also play a role in age-related hair regeneration [29].

### Msx2 deficiency leads to trans-epidermal differentiation of HFSC

**Fig. 4:**
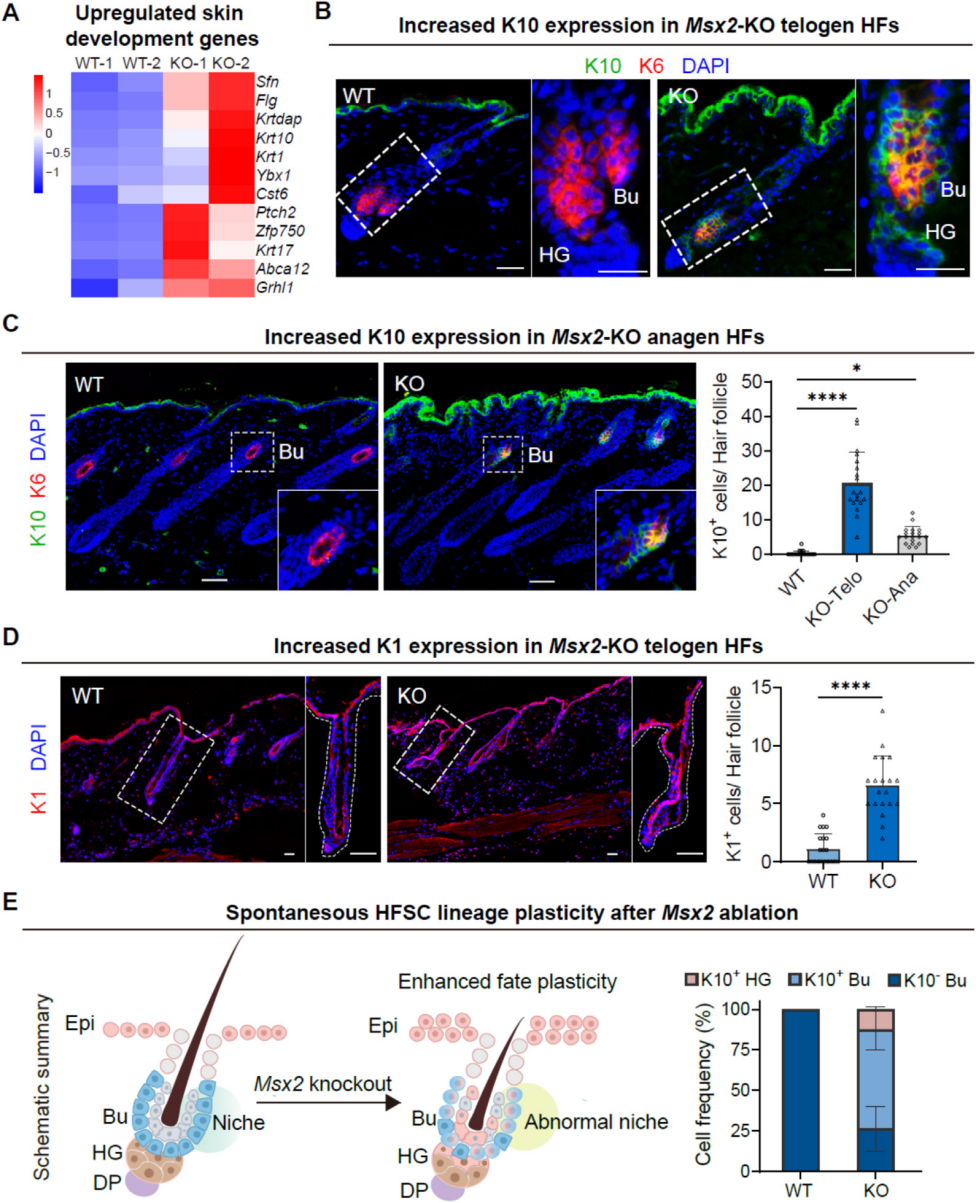
***Msx2*****loss promotes trans-epidermal HFSC differentiation.** (A) Heatmap of upregulated skin development genes as identified by RNA-seq analysis. (B) Immunofluorescence staining shows ectopic expression of K10 in *Msx2*-KO telogen bulge (Bu) and hair germ (HG). Scale bars, 30 μm. (C) Immunofluorescence staining shows ectopic expression of K10 in *Msx2*-KO anagen bulge (left) with quantification of positive cells (right). Scale bars, 30 μm. *N* = 3, ****p < 0.0001, *p < 0.05. (D) Immunofluorescence staining shows ectopic expression of K1 in *Msx2*-KO telogen HFs (left) with quantification of positive cells (right). Scale bars, 30 μm. *N* = 3, ****p < 0.0001. (E) Model of Msx2-mediated fate regulation (left) with quantification of epidermal marker cells in bulge (Bu) or hair germ (HG) compartments (right). *N* = 3. Epi, epidermis; DP, dermal papilla.

Following our examination of the transcriptional consequences of *Msx2* deficiency on HFSC, we systematically investigated the upregulated DEGs within the HFSC transcriptome (Fig. 2C). Our analysis uncovered a notable upregulation of genes linked to epidermal identity and differentiation, such as *Krt10*, *Krt1*, *Flg*, *Sfn* and *Krtdap* (Fig. 4A). Confirmation through immunofluorescence staining showed ectopic localization of K10 and K1 within the bulge region of mutant hair follicles (Fig. 4B and Fig. 4D). The persistence of these cells into the anagen phase suggested an early commitment to epidermal lineage differentiation (Fig. 4C).

Lineage plasticity represents a hallmark of damage-induced cellular responses, endowing stem cells with the flexibility to choose their fate. This phenomenon is characterized by the concurrent expression of transcription factors typical of both new and old lineages [30]. Upon further examination, we noted a predominant co-expression of K10 and K15 in HFSCs of the *Msx2*-KO mice, accompanied by aberrant expression of K10 in HG cells (Fig. 4B and E). This observation indicates that HFSCs remain arrested in a plastic state in the absence of *Msx2*. Moreover, we observed a remarkable rise in K10 expression in the epidermis of *Msx2*-KO mice (Fig. 4B and C), coinciding with the initiation of *Msx2* expression in the epidermis from the second hair follicle cycle (Fig. S4B). Prior research has implicated COL17A1 proteolysis in the loss of HFSC commitment to epidermal keratinocytes in the context of aging [31]. However, our results indicate that the altered fate of HFSCs in *Msx2*-deficient skin is independent of *Col17a1*, as no significant expression defects were detected (Fig. S2C). These results strongly suggest that the absence of *Msx2* redirects HFSC fate decisions and accelerates transepidermal differentiation in a manner not reliant on COL17A1.

To further elucidate the impact of Msx2 on epidermal differentiation, we performed RNA-seq on isolated EpdSCs from WT and *Msx2*-KO mice skin. GO enrichment analysis revealed a significant enrichment of genes involved in protein-DNA complex assembly, DNA replication, and chromatin assembly in KO EpdSCs, suggesting a heightened susceptibility to DNA damage accumulation (Fig. S4A). Moreover, *Msx2* expression in EpdSCs declined with age (Fig. S4C), consistent with reports that DNA damage can trigger differentiation as a protective mechanism [31], potentially explaining the observed enhancement in epidermal differentiation upon *Msx2* loss.

### Msx2 modulates HFSC activation by coordinating TGF-β and Wnt signaling

**Fig. 5:**
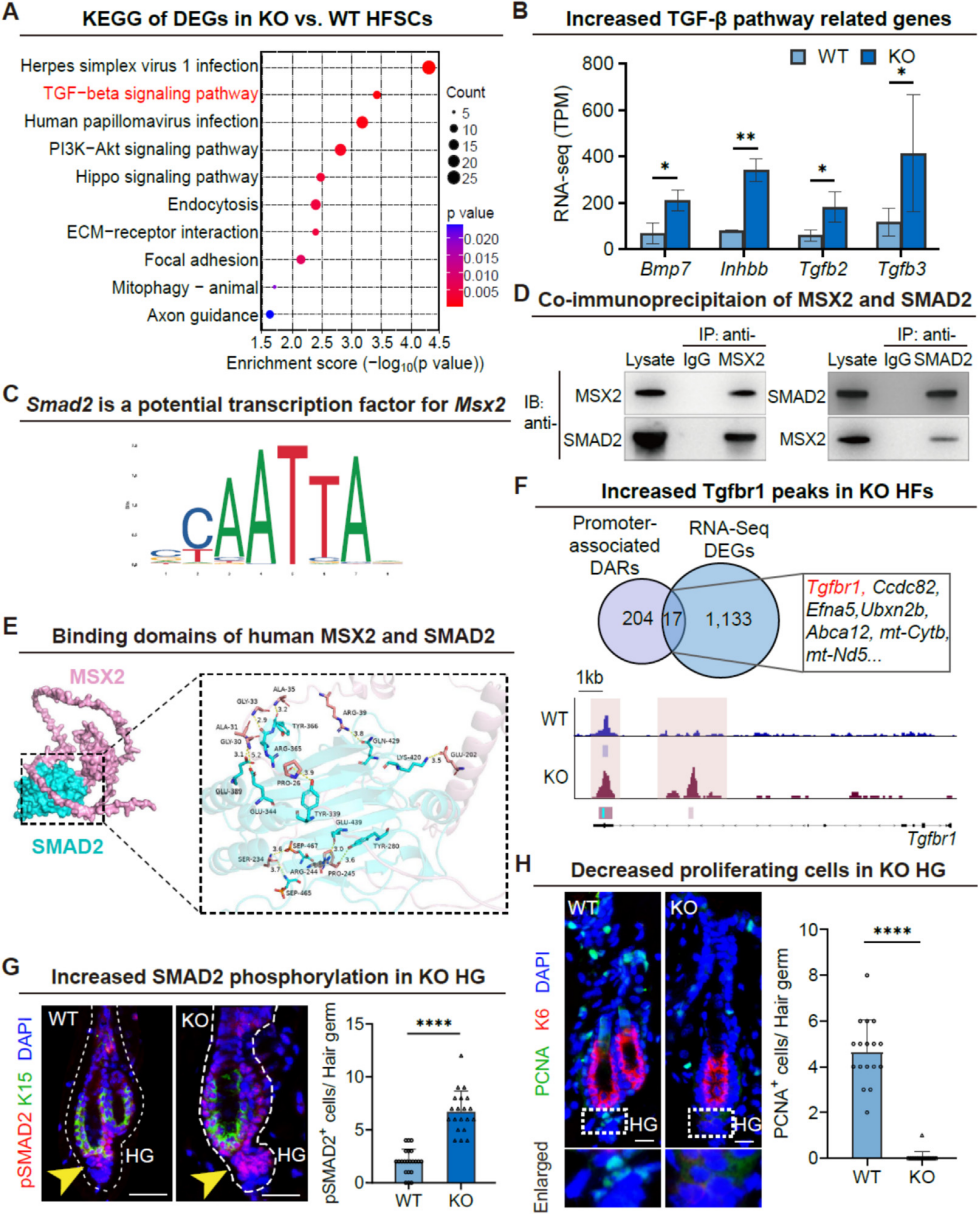
**Msx2 regulates HFSC activation and fate specification.** (A) KEGG pathway analysis shows the enrichment of TGF-β signaling pathway genes among DEGs. (B) Relative RNA expression levels of TGF-β pathway genes identified through RNA-seq analysis. *N* = 2, **p < 0.01, *p < 0.05. (C) Predicted *Smad2* binding sites in *Msx2* promoter. (D) Co-IP assays demonstrate a specific protein–protein interaction between MSX2 and SMAD2 in keratinocytes. (E) PyMOL modeling displays molecular docking between MSX2 and SMAD2. (F) Overlap between active promoter-associated differentially accessible regions (DARs) identified by ATAC-seq and DEGs from RNA-seq (top); representative example of chromatin accessibility at the *Tgfbr1* locus (bottom). (G) Immunofluorescence staining (left) with quantification (right) of pSMAD2 in telogen hair germ (HG). Scale bars, 30 μm. *N* = 3, ****p < 0.0001. (H) Immunofluorescence staining (left) with quantification (right) of PCNA in telogen HG. Scale bars, 30 μm. *N* = 3, ****p < 0.0001.

In light of the disruptions observed in the HFSC niche of *Msx2*-mutant hair follicles, we sought to determine whether these alterations influenced the signaling interactions between HG and DP cells, potentially affecting HFSC activation and lineage differentiation. To explore this, we conducted a comprehensive analysis of the Kyoto Encyclopedia of Genes and Genomes (KEGG) pathways and Ingenuity Pathway Analysis (IPA) corresponding to the DEGs identified in our study. Notably, genes associated with the TGF-β signaling pathway were significantly enriched and ranked among the top-represented gene groups (Fig. 5A). Concurrently, several hallmark genes of the TGF-β pathway were found to be upregulated in mutant hair follicles (Fig. 5B). Prior research has underscored the critical role of the TGF-β signaling pathway in HFSC activation, particularly by facilitating the crosstalk between HG and DP during the transition from telogen to anagen [32].

To investigate whether TGF-β signaling directly regulates Msx2, we utilized the Animal Transcription Factor Database (AnimalTFDB) v4.0 [33] and performed transcription factor binding site prediction to identify candidate regulators of the *Msx2* promoter. This analysis revealed potential binding sites for Smad family members, including Smad2 and its common mediator Smad4 (Fig. 5C). In addition, the JASPAR database predicted a highly confident binding site for Smad2 with Msx2, with a confidence level surpassing 97 %. Co-IP assays confirmed a physical interaction between MSX2 and SMAD2 proteins [15] (Fig. 5D), and molecular docking analysis revealed their likely binding domains (Fig. 5E).

To further determine how *Msx2* deletion affects chromatin dynamics, we performed ATAC-Seq on HFSCs from WT and KO mice. Among the DEGs, we identified 17 overlapping active promoter-associated differentially accessible regions (DARs), including a peak in the TGF-β1 receptor (*Tgfbr1*) locus that was enriched in KO mice (Fig. 5F). The genomic distribution of ATAC-seq peaks revealed that a substantial proportion were located in intergenic regions (Fig. S5C). Immunofluorescence staining results indicated that the phosphorylation of SMAD2 (pSMAD2), induced by TGF-β1 receptor signaling, was greatly accumulated in the HGs of mutants (Fig. 5G), consistent with premature SMAD2 activation and exacerbated TGF-β signaling prior to anagen onset.

Moreover, we noticed that promoter-associated DARs at inactive genes, suggesting repressive promoters, are genes involved in the β-catenin destruction complex and Wnt signalosome (Fig. S5D). LEF1, a canonical Wnt effector [34], was downregulated in both DP and HG compartments during telogen and anagen phases in *Msx2*-deficient HFs (Fig. S5A and Fig. S5B), suggesting impaired Wnt signaling. Consistently, proliferating cell nuclear antigen (PCNA) staining showed a significant reduction of proliferative cells in the mutant HG, indicative of HFSC quiescence (Fig. 5H).

Our results demonstrate that Msx2 functions as a critical modulator of HFSC activation by orchestrating the balance between TGF-β and Wnt signaling pathways. Loss of *Msx2* leads to increased chromatin accessibility at the *Tgfbr1* locus, elevated pSMAD2 levels in the HG, and upregulation of TGF-β signaling activity, suggesting that Msx2 constrains or fine-tunes TGF-β pathway activation during early telogen. Concurrently, Wnt signaling is dampened, as evidenced by reduced LEF1 expression and altered chromatin accessibility, contributing to the failure of HFSCs to exit quiescence. This dual dysregulation is accompanied by reduced HFSC proliferation and failure to initiate anagen, suggesting that Msx2 promotes HFSC activation by both limiting excessive TGF-β signaling and supporting Wnt-driven regenerative cues. Together, these findings position *Msx2* as a key transcriptional regulator that balances antagonistic cues to ensure timely HFSC activation and proper hair follicle regeneration.

### Msx2 modulates keratinocyte cytoskeletal organization, cell proliferation, and differentiation through the TGF-β signaling pathway

The TGF-β signaling pathway plays a crucial role in mediating EMT and remodeling of the ECM, processes that can enhance cancer cell invasion and metastasis [35]. Keratinocytes have been identified as a primary source of TGF-β1 in the epidermis [36]. Given our prior findings that *Msx2* deficiency results in heightened TGF-β signaling *in vivo*, we hypothesized that Msx2 may act as a suppressor of TGF-β1 expression in keratinocytes, thereby modulating downstream signaling and cellular behaviors.

**Fig. 6:**
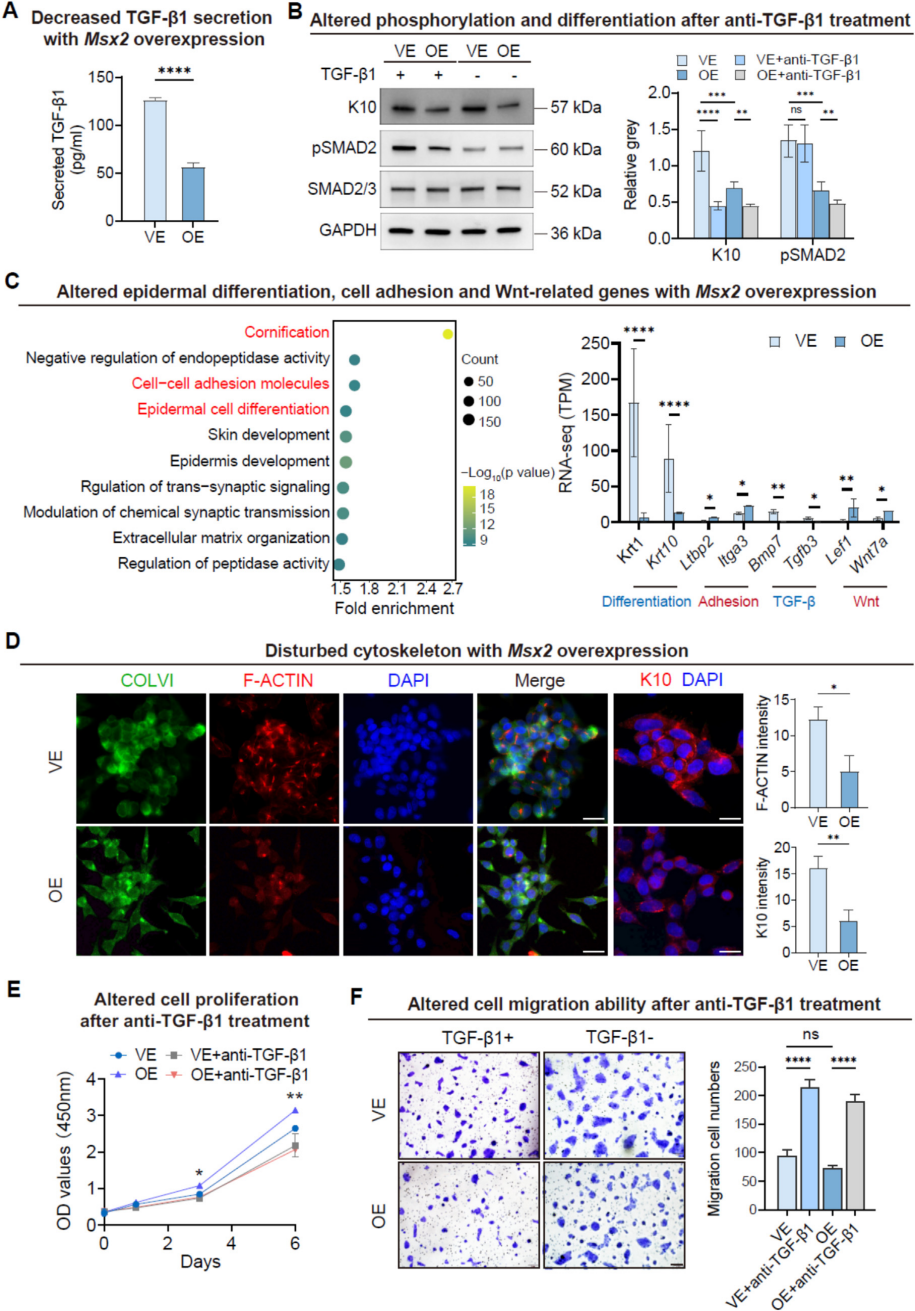
**Msx2 modulates cytoskeletal organization, proliferation, and differentiation via TGF-β signaling.** (A) ELISA analysis of TGF-β1 secretion from control (VE) and *Msx2*-overexpressing (OE) cells. *N* = 3, ****p < 0.0001. (B) Western blot (left) and statistical analysis (right) of indicated proteins in VE, OE, and TGF-β1-neutralized cells. *N* = 3, ****p < 0.0001, ***p < 0.001, **p < 0.01; ns, not significant. (C) KEGG analysis (left) and relative RNA expression levels of DEGs identified through RNA-seq analysis (right). *N* = 3, ****p < 0.0001, **p < 0.01, *p < 0.05. (D) Immunofluorescence staining (left) with quantification (right) of relative fluorescence intensity of cytoskeleton and differentiation markers. Scale bars, 50 μm. *N* = 3, **p < 0.01, *p < 0.05. (E) Proliferation assays with TGF-β1 neutralization. *N* = 3, **p < 0.01, *p < 0.05. (F) Transwell assays (left) and statistical analysis (right) comparing migration abilities in VE, OE, and TGF-β1-neutralized cells. Scale bars, 1 mm*. N* = 3, ****p < 0.0001; ns, not significant.

To validate this hypothesis, we first assessed the expression levels of TGF-β1 in keratinocytes overexpressing *Msx2* (Fig. S6A). Consistent with our hypothesis, we observed a marked reduction in TGF-β1 levels (Fig. 6A and Fig. S6B), accompanied by diminished phosphorylation of SMAD2 (Fig. 6B and Fig. S6C). Bulk RNA-seq of *Msx2*-overexpressing cells revealed enrichment of genes involved in epithelial organization, skin development, and cell–cell adhesion (Fig. 6C). Notably, this included downregulation of TGF-β signaling components and upregulation of Wnt pathway genes, further supporting our earlier mechanistic findings (Fig. 6C).

Additionally, the expression of the differentiation marker K10 was decreased (Fig. 6B–D), and cytoskeletal architecture appeared disrupted in *Msx2*-overexpressing cells (Fig. 6D). To determine whether TGF-β1 signaling contributes to the induction of cellular differentiation, we introduced a neutralizing antibody against TGF-β1 in normal cells. Blocking TGF-β1 resulted in significantly reduced SMAD2 phosphorylation and attenuated epidermal differentiation, mimicking the effect of *Msx2* overexpression (Fig. 6B and D). These findings suggest that Msx2 modulates keratinocyte differentiation, at least in part, through attenuation of autocrine TGF-β1 signaling.

Subsequently, we evaluated the proliferation and migration capabilities of cells in the different experimental groups. Proliferation assays indicated that Msx2 sustains a higher cell division rate in keratinocytes (Fig. 6E); however, neutralization of TGF-β1 resulted in decreased proliferation rates but enhanced cellular mobility (Fig. 6F), indicating possible changes in ECM adhesion dynamics. Collectively, these findings suggest that Msx2 promotes HFSC differentiation during hair follicle regeneration, while also regulating ECM organization and proliferative capacity via the TGF-β/Smad2 signaling axis.

## Discussion

In this study, we identify Msx2 as a key regulator of hair follicle cycling, particularly during the exogen phase. *Msx2*, predominantly expressed in HG and bulge cells, is essential for maintaining niche integrity and hair anchorage. Loss of *Msx2* results in cyclic alopecia, accompanied by defective expression of adhesion molecules and ECM components, leading to an impaired HFSC niche and premature detachment of the older bulge and club hair. Although HFSC identity remains intact, *Msx2* deficiency induces aberrant activation and trans-epidermal differentiation, as evidenced by upregulation of K10 and K1 and elevated apoptosis within the bulge. Transcriptomic analysis revealed overactivation of the TGF-β signaling pathway, characterized by increased SMAD2 phosphorylation during early telogen. Mechanistically, MSX2 physically interacts with SMAD2, as demonstrated by Co-IP and in vitro validation, implicating it in the modulation of TGF-β-driven differentiation and adhesion maintenance. Concurrently, suppression of Wnt signaling in KO hair follicles may further compromise HFSC quiescence and niche stability, collectively contributing to impaired regenerative capacity.

Exogen depends on its epithelial microenvironment, where the shedding of club hairs is regulated by complex molecular interactions [4]. Before shedding, the loosened exogen hair may be passively retained for a period, a phenomenon that bears similarities to the desquamation of terminally differentiated corneocytes from the stratum corneum [5]. Here, we employed RNAscope and RNA-Seq analyses to identify the dynamic expression profile of *Msx2* in hair follicles, confirming its critical role in anchoring the bulge. Mechanistically, our analyses revealed changes in ECM components (COLVI, POSTN, FBLN-1, and ANGPTL2) surrounding the HFSC niche, leading to weakened intercellular connections in *Msx2*-KO mice during the telogen and exogen phases. These modifications may promote fiber release through terminal differentiation or proteolytic cleavage [7]. Our findings highlight the intricate molecular processes governing hair shedding and the critical role of the epithelial microenvironment in exogen.

Prior studies have identified bulge structure defects in various mouse models, such as *Foxc1*-cKO and *Lhx2*-cKO mice, as well as aged mice [21], [37]. While Sox9, Lhx2, and Foxc1 preserve HFSC identity and quiescence via distinct mechanisms, our findings highlight Msx2 as a niche modulator that maintains HFSC homeostasis by sustaining cell–ECM adhesion, rather than directly governing stemness. Specifically, *Sox9* regulates the Wnt/BMP signaling balance, *Lhx2* maintains stem cell potential through chromatin remodeling, and *Foxc1* reinforces niche signaling by activating *Nfatc1* during the telogen-to-anagen transition [20], [37], [38]. Whether Msx2 shares regulatory features with these factors or operates through tissue-specific mechanisms remains to be further investigated. Moreover, COLVI is abundantly expressed in the telogen bulge region [39], yet restoring individual ECM components appears insufficient to rescue hair growth [25]. These findings underscore the necessity of coordinated niche architecture and signaling for sustaining long-term follicular stability.

Lineage plasticity has emerged as a pivotal feature of the stem cell stress response [20], [30]. More specifically, HFSCs are primarily responsible for promoting hair growth; however, a subset of these quiescent HFSCs can undergo trans-epidermal differentiation in response to tissue injury, transforming into epidermal keratinocytes. This ability highlights their role in epidermal regeneration and wound healing [30]. Previously, alterations in the proteolysis of COL17A1 have been shown to induce physiological aging in HFSCs, leading to the loss of stemness characteristics and commitment to terminal epidermal differentiation [31]. Recent research has demonstrated that local retinoic acid signaling is transiently diminished in HFSCs when niche architecture is compromised, enhancing lineage plasticity and promoting fate shifts via cues such as Wnt and BMP [30]. In our study, *Msx2*-KO bulge cells exhibited a shift toward epidermal lineage, marked by increased K10 and reduced LEF1 expression. Pathway analyses illustrate pronounced alterations in both Wnt/β-catenin and TGF-β pathways within *Msx2*-KO hair follicles. Notably, similar changes in these pathways have also been reported in AGA patients [40]. During the telogen-to-anagen transition, both Wnt and TGF-β signaling pathways are key regulators that modulate BMP thresholds [32]. Exogenous TGF-β1 injections or overexpression of active TGF-β1 inhibit entry into anagen [41]. *In vitro* studies have reported that DP cells from the scalps of patients with AGA secrete paracrine factors, including TGF-β1, which regulate hair follicle keratinocyte activity [42]. Consistently, elevated TGF-β1 protein levels in balding scalp regions have been associated with early DP vascular degeneration and hair follicle miniaturization [43]. In line with these findings, *Msx2* overexpression in our model inhibited TGF-β/Smad signaling, suppressed epidermal differentiation, and partially restored LEF1 expression, suggesting that TGF-β signaling may antagonize Wnt activity to promote epidermal fate. Although this indicates potential crosstalk between the TGF-β and Wnt pathways, direct evidence linking Wnt inhibition to epidermal specification remains limited. An important consideration is whether exogenous TGF-β supplementation could rescue the ECM defects and defective anchoring phenotype observed in *Msx2*-deficient follicles. However, both our data and prior reports indicate that TGF-β signaling is already elevated under *Msx2* deficiency and that excess TGF-β activity suppresses anagen initiation and contributes to follicle miniaturization in AGA. Thus, additional TGF-β input is unlikely to normalize ECM deposition and may instead exacerbate follicular instability. A more promising therapeutic approach may lie in restoring balanced TGF-β/Smad2 signaling. Future studies employing genetic or pharmacologic perturbation of TGF-β1/Smad2, coupled with single-cell transcriptomics and lineage tracing, will be required to directly test whether normalization of this pathway can re-establish ECM organization and stabilize hair anchoring.

In skin, Msx2 has been shown to support epithelial competency during WIHN [13]. Our findings suggest that ectopic expression of epidermal markers in mutant HFSCs reflects a shift toward trans-epidermal differentiation. The proximity of the anagen bulge to the epidermis implies that HFSCs lacking *Msx2* fail to differentiate into hair follicles, instead adopting markers indicative of epidermal differentiation. This transition leads to their development into keratinized corneocytes that migrate towards the skin surface for eventual desquamation, resulting in progressive hair follicle miniaturization, thinning hair, and consequent hair loss. Consistent with previous studies, these observations suggest that the regulation of epidermal differentiation and desquamation may involve processes similar to those observed in the maturation and shedding of club fibers [8]. Specifically, the proteolysis of cell adhesion structures plays a crucial role in desquamation by reducing cell–cell adhesion, thereby facilitating the shedding of corneocytes. Clinically, conditions such as severe dandruff and seborrheic dermatitis, which exhibit alterations in epidermal desquamation, are often associated with changes in hair shedding [44]. Therefore, the role of Msx2 in regulating both exogen and epidermal differentiation warrants further investigation. Future investigations employing follicular epithelial-specific *Msx2*-KO models will be instrumental in delineating its cell type–specific functions. Complementary *Msx2* overexpression models can further illuminate its role in modulating the balance between epidermal and follicular lineage commitment. These approaches will deepen our understanding of Msx2 in regulating HFSC fate decisions and its impact on hair follicle maintenance and disorders like AGA.

## Conclusion

Our study reveals Msx2 as a central regulator of the exogen phase, safeguarding HFSC niche integrity, maintaining bulge architecture, and coordinating lineage commitment during hair follicle regeneration. By modulating the balance between TGF-β and Wnt signaling, Msx2 ensures proper HFSC activation and prevents premature epidermal differentiation and hair shedding. These findings not only deepen mechanistic insight into the cellular and molecular orchestration of the hair cycle but also implicate *Msx2* dysfunction in the pathogenesis of hair loss disorders, positioning it as a promising therapeutic target for regenerative dermatology.

## Compliance with ethics requirement

All animal procedures were conducted in accordance with protocol #9473 approved by the Institutional Animal Care and Utilization Committee (IACUC) of the University of Southern California (USC, Los Angeles, CA, USA).

## Declaration of competing interest

The authors declare that they have no known competing financial interests or personal relationships that could have appeared to influence the work reported in this paper.
