# Supplementary material for: Roles of Msx2 in exogen control: modulating the stem cell niche during the transition from hair shedding to regeneration
Source: J Adv Res. 2025 Sep 23;84:345–59. doi: 10.1016/j.jare.2025.09.040 (PMC13227282; doi:10.1016/j.jare.2025.09.040)
Supplement: Supplementary Data 1 [file mmc1.pdf]

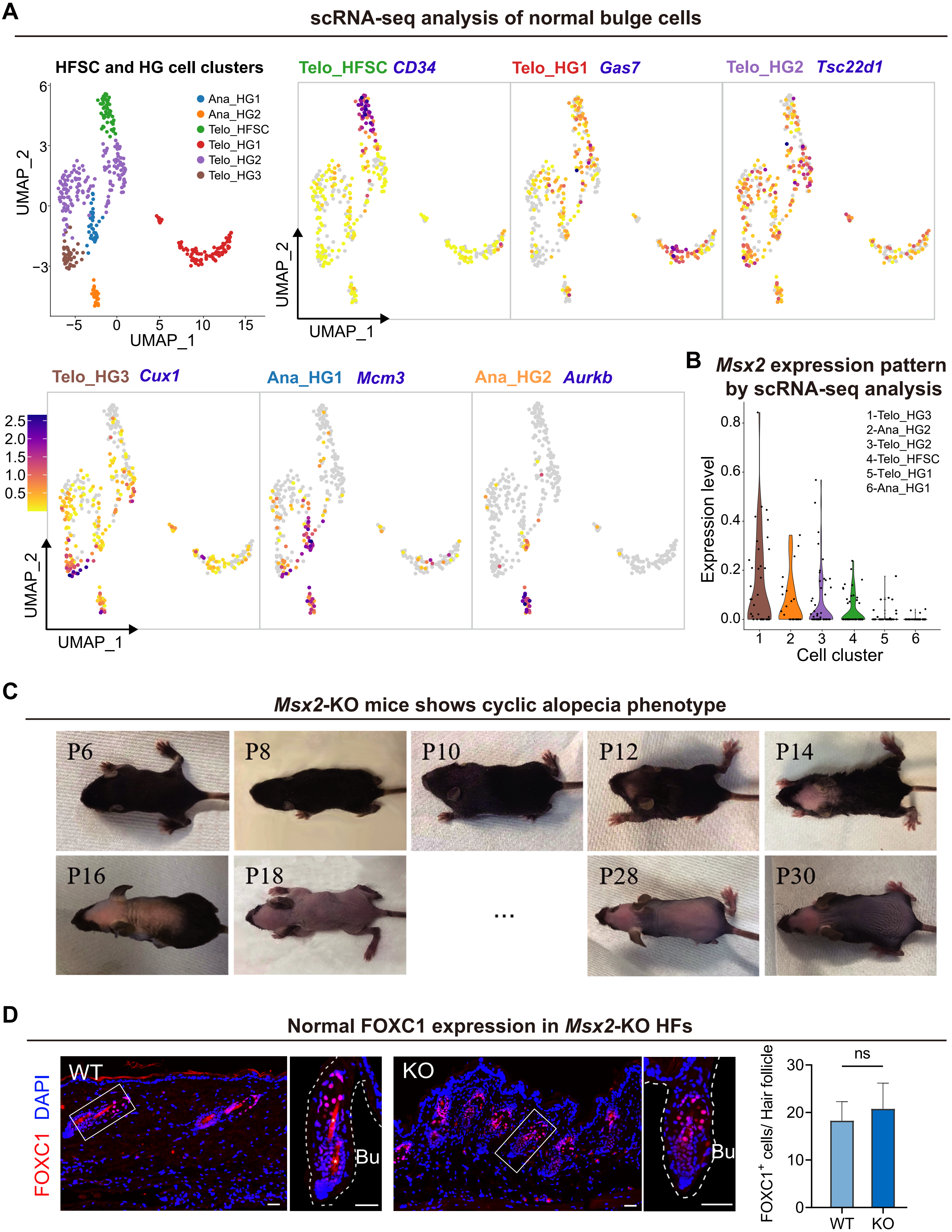
A
scRNA-seq analysis of normal bulge cells
HFSC and HG cell clusters
Ana_HG1
Ana_HG2
Telo_HFSC
Telo_HG1
Telo_HG2
Telo_HG3
UMAP_1
UMAP_2
Telo_HFSC CD34
Telo_HG1 Gas7
Telo_HG2 Tsc22d1
Telo_HG3 Cux1
Ana_HG1 Mcm3
Ana_HG2 Aurkb
B
Msx2 expression pattern by scRNA-seq analysis
1-Telo_HG3
2-Ana_HG2
3-Telo_HG2
4-Telo_HFSC
5-Telo_HG1
6-Ana_HG1
Expression level
Cell cluster
C
Msx2-KO mice shows cyclic alopecia phenotype
P6
P8
P10
P12
P14
P16
P18
...
P28
P30
D
Normal FOXC1 expression in Msx2-KO HFs
FOXC1 DAPI
WT
KO
Bu
FOXC1+ cells/ Hair follicle
ns
WT
KO
