# Supplementary material for: Roles of Msx2 in exogen control: modulating the stem cell niche during the transition from hair shedding to regeneration
Source: J Adv Res. 2025 Sep 23;84:345–59. doi: 10.1016/j.jare.2025.09.040 (PMC13227282; doi:10.1016/j.jare.2025.09.040)
Supplement: Supplementary Data 2 [file mmc2.pdf]

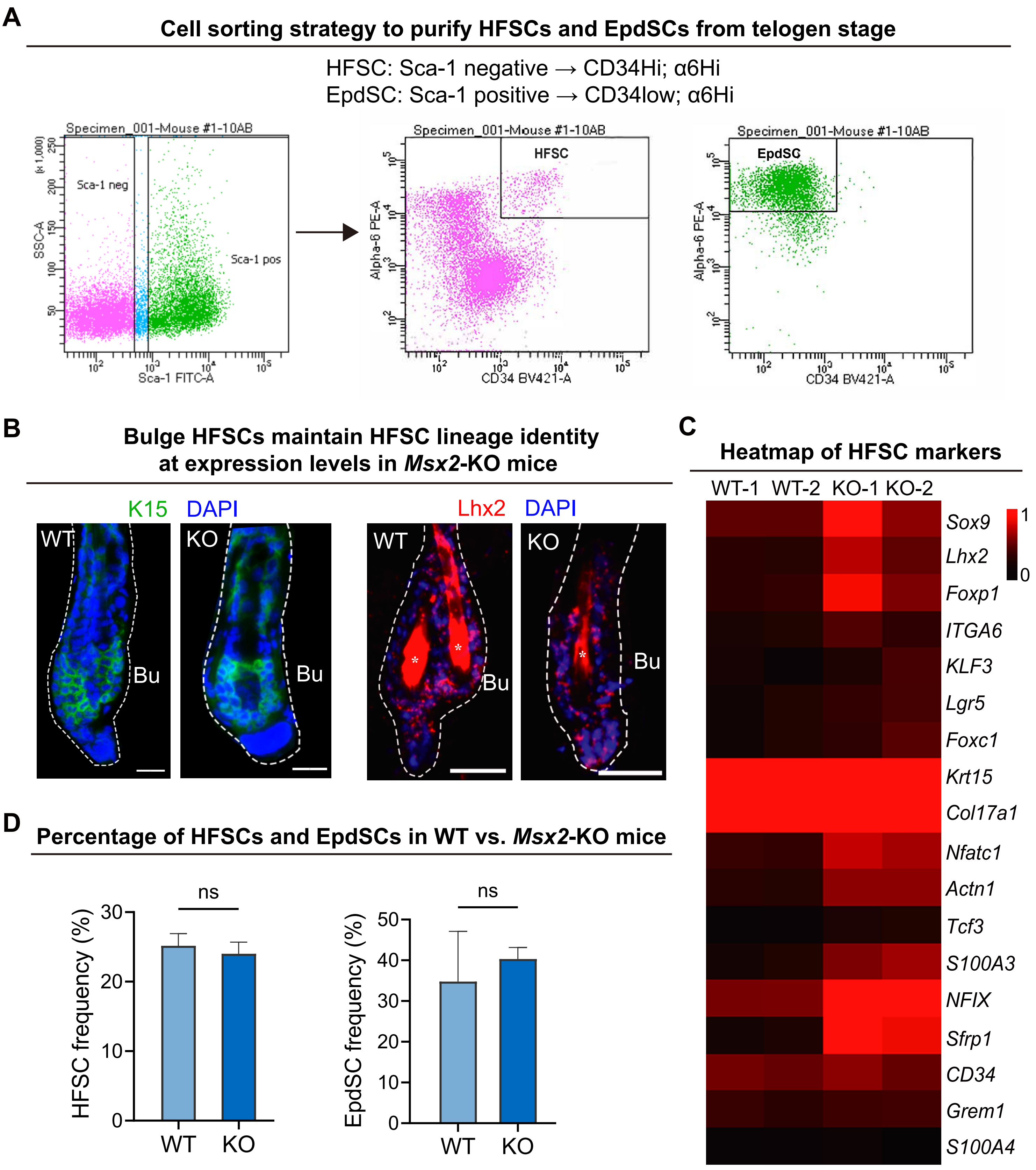
A
Cell sorting strategy to purify HFSCs and EpdSCs from telogen stage
HFSC: Sca-1 negative → CD34Hi; α6Hi
EpdSC: Sca-1 positive → CD34low; α6Hi
Specimen_001-Mouse #1-10AB
Sca-1 neg
Sca-1 pos
SSC-A
(x 1,000)
Sca-1 FITC-A
HFSC
Alpha-6 PE-A
CD34 BV421-A
EpdSC
B
Bulge HFSCs maintain HFSC lineage identity at expression levels in Msx2-KO mice
K15 DAPI
WT
KO
Bu
Lhx2 DAPI
C
Heatmap of HFSC markers
WT-1 WT-2 KO-1 KO-2
Sox9
Lhx2
Foxp1
ITGA6
KLF3
Lgr5
Foxc1
Krt15
Col17a1
Nfatc1
Actn1
Tcf3
S100A3
NFIX
Sfrp1
CD34
Grem1
S100A4
1
0
D
Percentage of HFSCs and EpdSCs in WT vs. Msx2-KO mice
ns
HFSC frequency (%)
EpdSC frequency (%)
WT
KO
