# Supplementary material for: Roles of Msx2 in exogen control: modulating the stem cell niche during the transition from hair shedding to regeneration
Source: J Adv Res. 2025 Sep 23;84:345–59. doi: 10.1016/j.jare.2025.09.040 (PMC13227282; doi:10.1016/j.jare.2025.09.040)
Supplement: Supplementary Data 3 [file mmc3.pdf]

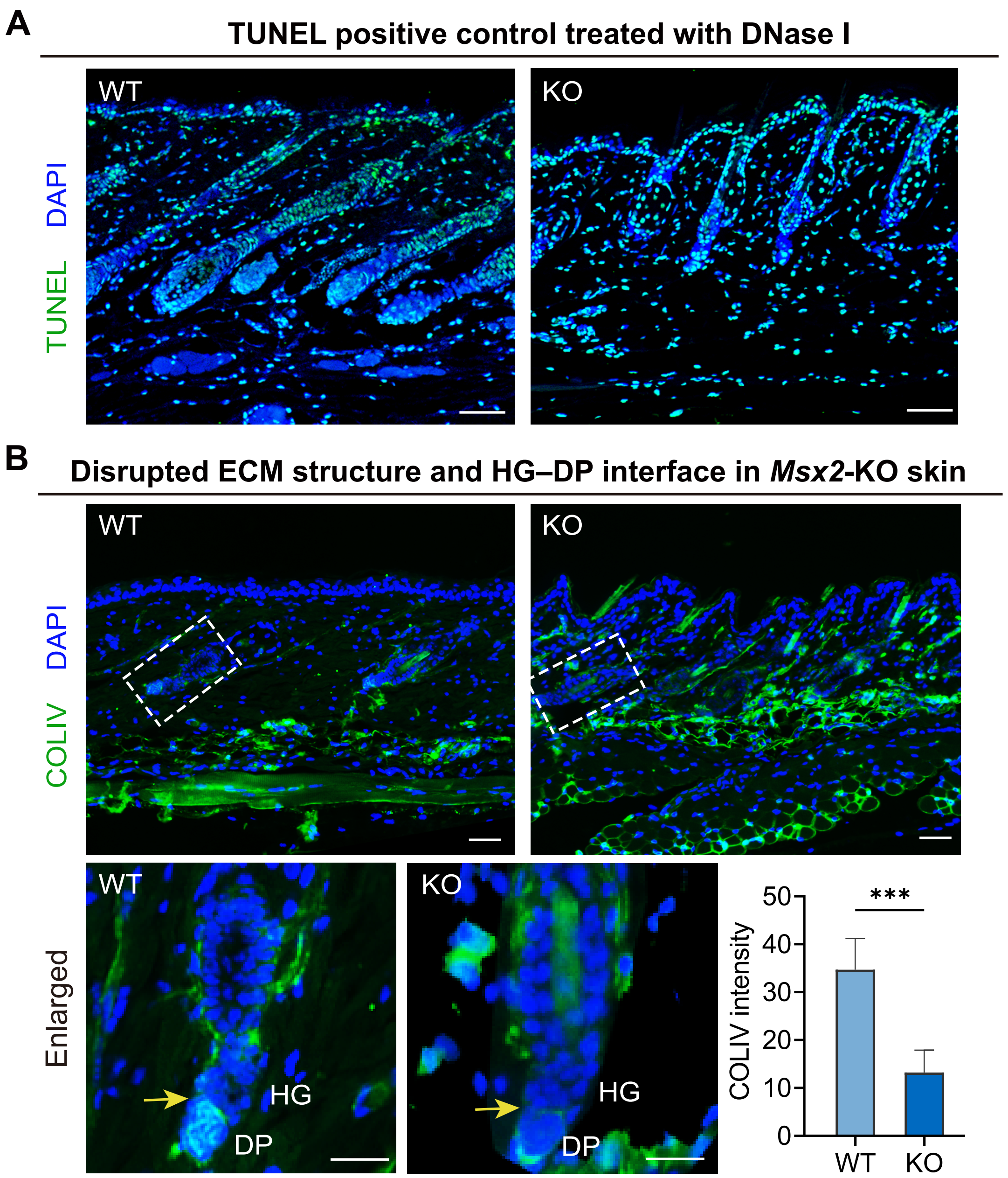
A
TUNEL positive control treated with DNase I
WT
KO
TUNEL DAPI
B
Disrupted ECM structure and HG–DP interface in Msx2-KO skin
WT
KO
COLIV DAPI
Enlarged
WT
HG
DP
KO
HG
DP
COLIV intensity
50
40
30
20
10
0
***
WT
KO
