# Supplementary material for: Roles of Msx2 in exogen control: modulating the stem cell niche during the transition from hair shedding to regeneration
Source: J Adv Res. 2025 Sep 23;84:345–59. doi: 10.1016/j.jare.2025.09.040 (PMC13227282; doi:10.1016/j.jare.2025.09.040)

A

Gene Ontology analysis of DEGs in EpdSCs from *Msx2*-KO vs. WT skin

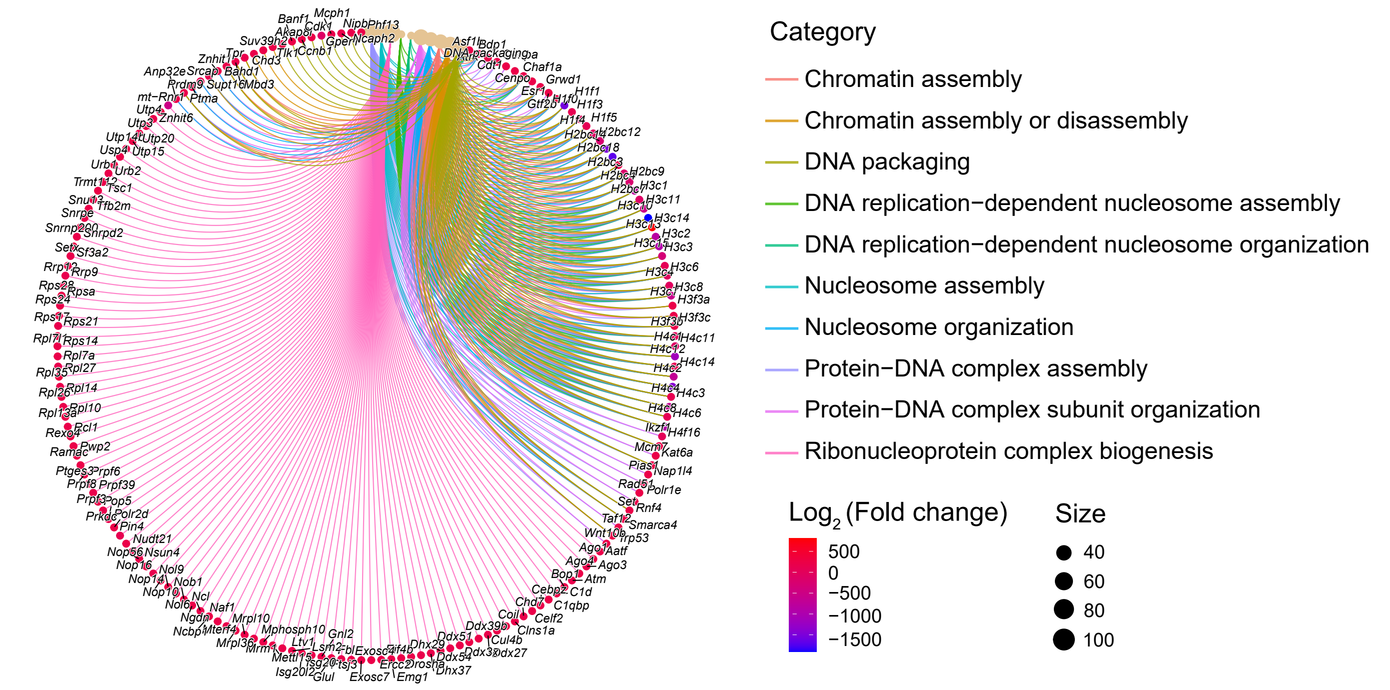

B

Msx2 is expressed in EpdSCs

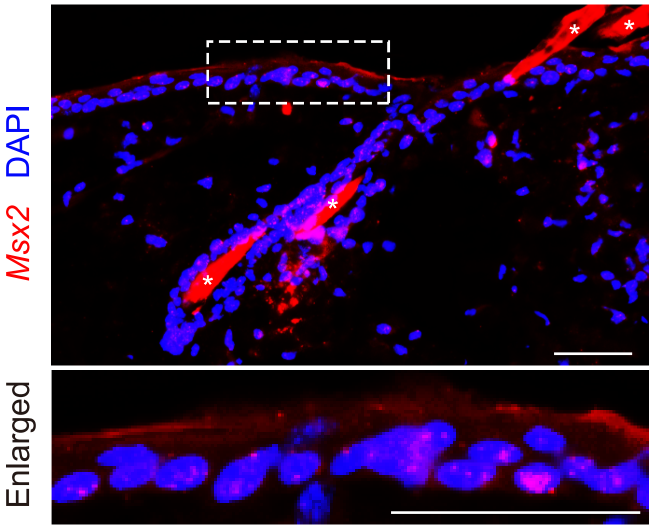

C

Msx2 is downregulated in aging EpdSCs

Gene	Fold change (2yrs/2mths)	EpdSC 2mths (RPKM)	EpdSC 2yrs (RPKM)
Msx2	-2.1875	6.2236	2.8451

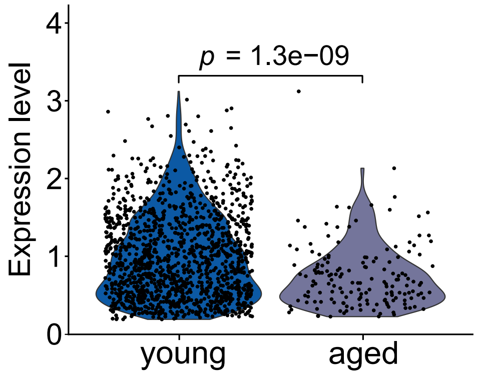

Supplement: Supplementary Data 4 [file mmc4.pdf]
