# Supplementary material for: Roles of Msx2 in exogen control: modulating the stem cell niche during the transition from hair shedding to regeneration
Source: J Adv Res. 2025 Sep 23;84:345–59. doi: 10.1016/j.jare.2025.09.040 (PMC13227282; doi:10.1016/j.jare.2025.09.040)
Supplement: Supplementary Data 5 [file mmc5.pdf]

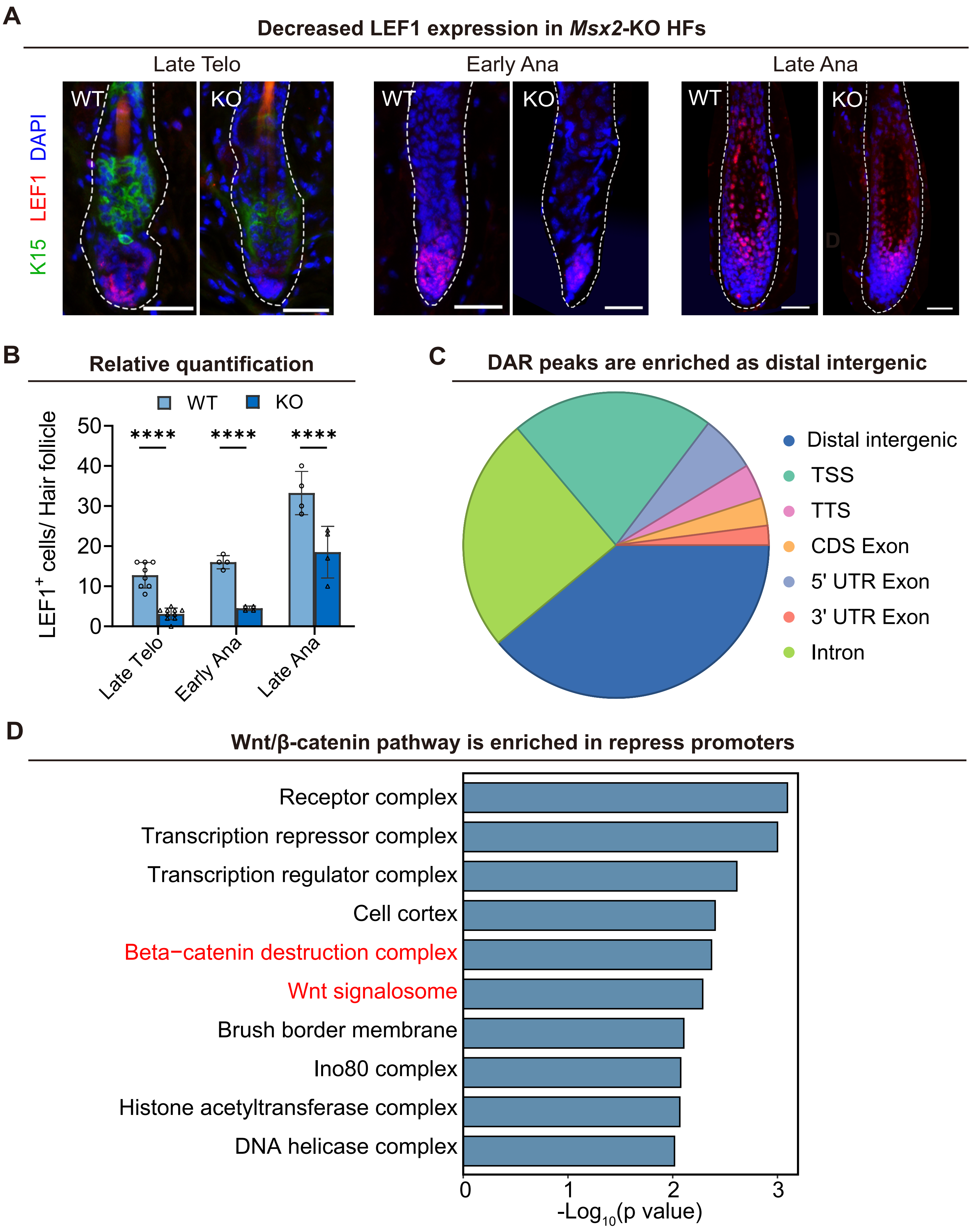
A
Decreased LEF1 expression in Msx2-KO HFs
Late Telo
Early Ana
Late Ana
WT
KO
K15
LEF1
DAPI
B
Relative quantification
WT
KO
****
LEF1+ cells/ Hair follicle
0
10
20
30
40
50
Late Telo
Early Ana
Late Ana
C
DAR peaks are enriched as distal intergenic
Distal intergenic
TSS
TTS
CDS Exon
5' UTR Exon
3' UTR Exon
Intron
D
Wnt/β-catenin pathway is enriched in repress promoters
Receptor complex
Transcription repressor complex
Transcription regulator complex
Cell cortex
Beta−catenin destruction complex
Wnt signalosome
Brush border membrane
Ino80 complex
Histone acetyltransferase complex
DNA helicase complex
0
1
2
3
-Log10(p value)
