# Supplementary figures and images for: Roles of Msx2 in exogen control: modulating the stem cell niche during the transition from hair shedding to regeneration
Source: J Adv Res. 2025 Sep 23;84:345–59. doi: 10.1016/j.jare.2025.09.040 (PMC13227282; doi:10.1016/j.jare.2025.09.040)

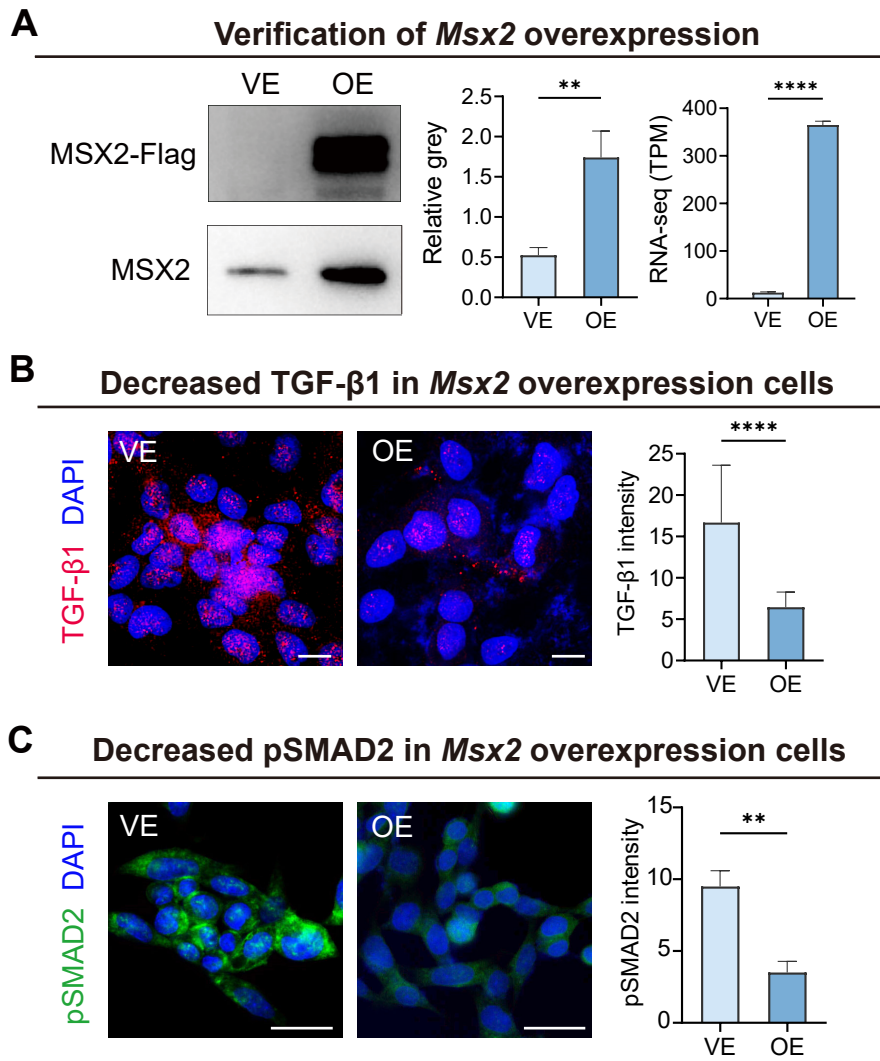

Supplement: Supplementary Data 6 [file mmc6.pdf]
